# A Limited Number of Antibody Specificities Mediate Broad and Potent Serum Neutralization in Selected HIV-1 Infected Individuals

**DOI:** 10.1371/journal.ppat.1001028

**Published:** 2010-08-05

**Authors:** Laura M. Walker, Melissa D. Simek, Frances Priddy, Johannes S. Gach, Denise Wagner, Michael B. Zwick, Sanjay K. Phogat, Pascal Poignard, Dennis R. Burton

**Affiliations:** 1 Department of Immunology and Microbial Science and IAVI Neutralizing Antibody Center, The Scripps Research Institute, La Jolla, California, United States of America; 2 International AIDS Vaccine Initiative, New York, New York, United States of America; 3 IAVI AIDS Vaccine Design and Development Laboratory, Brooklyn, New York, United States of America; 4 Ragon Institute of MGH, MIT, and Harvard, Boston, Massachusetts, United States of America; University of Zurich, Switzerland

## Abstract

A protective vaccine against HIV-1 will likely require the elicitation of a broadly neutralizing antibody (bNAb) response. Although the development of an immunogen that elicits such antibodies remains elusive, a proportion of HIV-1 infected individuals evolve broadly neutralizing serum responses over time, demonstrating that the human immune system can recognize and generate NAbs to conserved epitopes on the virus. Understanding the specificities that mediate broad neutralization will provide insight into which epitopes should be targeted for immunogen design and aid in the isolation of broadly neutralizing monoclonal antibodies from these donors. Here, we have used a number of new and established technologies to map the bNAb specificities in the sera of 19 donors who exhibit among the most potent cross-clade serum neutralizing activities observed to date. The results suggest that broad and potent serum neutralization arises in most donors through a limited number of specificities (1–2 per donor). The major targets recognized are an epitope defined by the bNAbs PG9 and PG16 that is associated with conserved regions of the V1, V2 and V3 loops, an epitope overlapping the CD4 binding site and possibly the coreceptor binding site, an epitope sensitive to a loss of the glycan at N332 and distinct from that recognized by the bNAb 2G12 and an epitope sensitive to an I165A substitution. In approximately half of the donors, key N-linked glycans were critical for expression of the epitopes recognized by the bNAb specificities in the sera.

## Author Summary

The development of an immunogen that elicits antibodies that neutralize a wide range of global circulating HIV-1 isolates is a major goal of HIV-1 vaccine research. Unfortunately, even the most promising antibody-based vaccine candidates have only induced NAb responses that neutralize a limited number of these strains. However, recent studies have demonstrated that broad and potent NAb responses develop in the sera of a subset of HIV-1 infected individuals, and studying the nature of these responses may provide clues for the design of new vaccine immunogens. Here, we show that the broad neutralization in the sera of most of the individual donors that we studied can be associated with single or a small number of specificities. Across the donor panel, broad neutralization appears associated with 4–5 principal specificities.

## Introduction

The hallmark of most successful anti-viral vaccines is the ability to induce neutralizing antibodies [1], [2], [3], [4]. For HIV-1, NAbs have been shown to provide protection against viral challenge in non-human primate models [5], [6], [7], [8], [9], [10], [11], [12], [13], suggesting that a vaccine capable of inducing similar types of antibodies would provide benefit upon exposure to the virus. However, due to the extraordinary genetic diversity of the HIV-1, a successful vaccine will require the induction of antibodies that neutralize a wide spectrum of global circulating viral isolates, i.e. broadly neutralizing antibodies (bNAbs) [14]. Unfortunately, the development of an immunogen capable of eliciting bNAbs has not been met with success to date. Importantly, although NAbs generated during natural HIV-1 infection usually target immunodominant variable regions of the virus, recent studies have shown that 10–30% of infected individuals develop moderate to broadly neutralizing sera [15], [16], [17], [18]. These individuals are of considerable interest from a vaccine standpoint; understanding the antibody specificities that mediate potent cross-clade serum neutralizing activity may illuminate potential targets for HIV-1 immunogen design. In addition, knowledge of the epitopes targeted by the bNAbs can assist in the design of reagents, “baits”, to facilitate the isolation of broadly neutralizing monoclonal antibodies (bnMAbs) from these donors. BnMAbs can be used in molecular studies to help direct vaccine design [19], [20], [21].

Several studies have previously been performed to systematically analyze the NAb specificities in HIV-1 positive sera displaying varying degrees of neutralization breadth and potency [15], [16], [17], [18], [22], [23], [24]. In all of these studies, a series of complementary methods, such as selective removal of certain antibody specificities using antigen-coated beads, inhibition of neutralizing activity using linear peptides, and the use of chimeric viruses displaying specific epitopes, were used to define the epitopes targeted by NAbs in broadly neutralizing sera. Although the breadth of serum neutralization could rarely be mapped exclusively to a single epitope, several sera appeared to contain CD4bs and co-receptor binding site (CRbs)-specific antibodies that contributed to the overall breadth of serum neutralizing activity [16], [17], [22], [25]. In a minority of cases, sera were found to contain NAbs to the membrane-proximal external region (MPER) [16], [17], [23]. Arguably, one of the most significant results from these studies was that a substantial fraction of the serum NAbs appeared to target unidentified viral epitopes. Considering that most of the reagents used for characterization were based on monomeric gp120 and linear peptides, one possibility here is that the serum neutralization breadth is mediated by NAbs that recognize quaternary epitopes preferentially expressed on trimeric Env. Two recently described broad and potent NAbs, PG9 and PG16, fall into this category [26].

An important question that has arisen from serum studies concerns the number of NAb specificities that mediate broad serum neutralization. A few scenarios are possible; broad serum neutralization could be mediated by a very large number of neutralizing antibodies with limited breadth [27], a few relatively broad and potent neutralizing antibody specificities, or a single, extraordinarily broad and potent, neutralizing antibody specificity. Although these scenarios are not mutually exclusive, the latter two are more attractive in terms of vaccine design, as it appears far more practical to focus the immune response on a small number of conserved epitopes with a vaccine rather than on a large number of more variable epitopes.

In a previous study, we screened sera from approximately 1,800 HIV-1 infected donors from Thailand, Australia, the United Kingdom, the United States, and several sub-Saharan African countries for neutralizing activity and identified donors who exhibit among the most broad and potent neutralizing serum activity observed to date [15]. The top 1% of samples screened, designated “elite neutralizers”, displayed particularly potent serum neutralizing activity against a cross-clade pseudovirus panel. These donors are valuable for understanding the development of broad responses and for the isolation of broad and potent neutralizing monoclonal antibodies. Notably, PG9 and PG16 were isolated from an individual who ranked in the top 5% of donors screened [26]. In this study, we have used a number of established and new techniques to map the broadly neutralizing antibody specificities in the serum of individuals who were ranked in the top 5% of neutralizers identified in our previous study, including elite neutralizers. Importantly, since many of our approaches rely on the use of functional assays, we defined the epitopes recognized by the broadly neutralizing serum antibodies in the context of the native trimer. Our results demonstrate that the broad neutralization in the sera of most of the individual donors can be associated with single or a small number of specificities. Across the donor panel, broad neutralization appears associated with 4–5 principal specificities.

## Results

### Serum neutralizing activity and demographics of selected donors

A total of 19 volunteers from diverse HIV-1 epidemics were characterized. Of the 19 volunteers, 63% were female, 26% male and 11% unknown. The median age of all volunteers was 38 with a median CD4 count of 414 and median log viral load of 4.07. All volunteers were infected for at least 3 years. Of the 19 donors analyzed, 14 ranked in the top 1% of neutralizers identified in our previous report (elite neutralizers), and the remaining 5 ranked in the top 5% of neutralizers. Figure S1 shows the serum neutralization profiles of the selected donors. Serum neutralization was assessed using an indicator cross-clade pseudovirus panel that has previously been shown to be predictive of neutralization breadth and potency over a larger number of isolates [15].

### Recombinant gp120 and/or gp140-reactive antibodies mediate broad serum neutralization in 70% of donors

**Figure 1:**
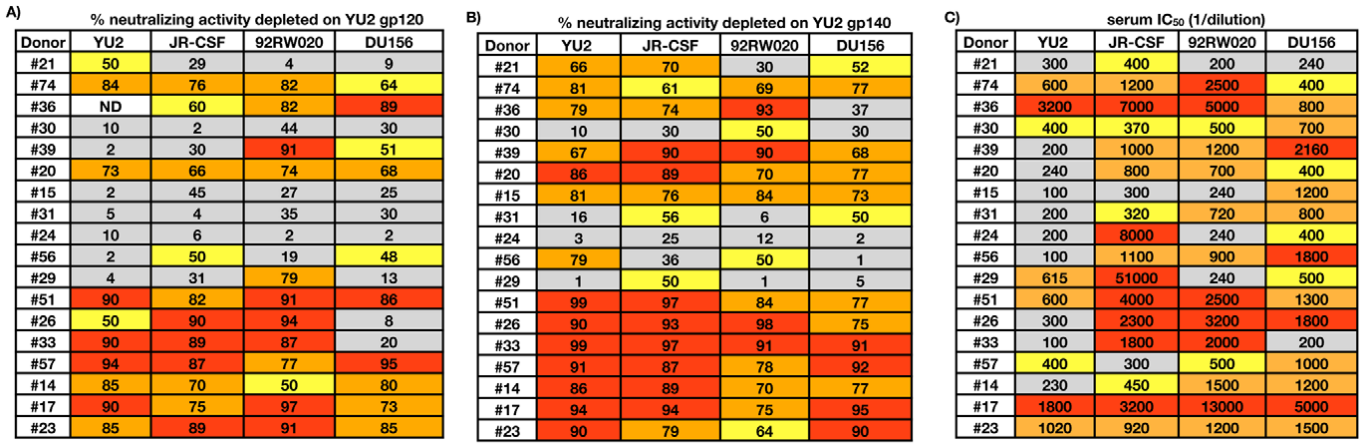
Serum adsorptions with recombinant Env proteins. Sera were adsorbed with YU2 gp120 or gp140-coupled beads or blank control beads and then tested for neutralizing activity against a cross-clade pseudovirus panel using TZM-bl target cells. A) Serum neutralizing activity depleted on YU2 gp120. B) Serum neutralizing activity depleted on YU2 gp140. Percent neutralizing activity depleted on YU2 gp120 or YU2 gp140 was calculated using the equation = (1−(IC_50 blank beads_/IC_50 antigen-coated beads_))*100. Boxes are color coded as follows: Gray, 0–45%, yellow, 45–65%; orange, 65–85%; red, 85–100%. C) Serum neutralizing activity against the selected cross-clade pseudovirus panel. Serum IC_50_ titers are color coded as follows: gray squares, 1∶300>IC50≥1∶100, yellow squares, 1∶500>IC50≥1∶300; orange squares, 1∶1500>IC50≥1∶500, red squares, IC50≥1∶1500.

As a first approach, we used a previously described serum adsorption method to determine whether the NAb specificities in these sera would react with recombinant monomeric gp120 [17]. Eighteen sera, which all neutralized HIV-1 YU2, were adsorbed with recombinant YU2 gp120 coupled beads or blank control beads (donor #37 was excluded from the analysis because the plasma did not neutralize YU2). After confirming depletion efficiencies by ELISAs, which showed that all detectable gp120-binding antibodies had been removed (Figure S2), the adsorbed fractions were tested for neutralizing activity against a cross-clade pseudovirus panel (Figure 1A). For ten donors (#74, #36, #20, #51, #26, #33, #57, #17, #14, and #23), most of the broad serum neutralizing activity was removed after gp120 adsorption, indicating that the serum neutralization breadth could be attributed to gp120-reactive NAbs. In contrast, for the remaining eight donors (#21, #30, #39, #15, #31, #24, #56, #29), a large proportion of the broad neutralizing serum activity was retained after removal of the YU2 gp120-specific Abs, suggesting the presence of NAbs that recognize epitopes that are not expressed on recombinant YU2 gp120. Based on these results, we next sought to determine whether the NAb specificities in the serum would react with a recombinant trimerized Env protein. The YU2 gp140-foldon trimer was chosen for these studies because it has been well characterized structurally and antigenically [28], [29], [30], [31], [32], [33], and it has been previously used for the isolation of NAbs from HIV-1 infected patients [27]. Using the same method as for gp120, we adsorbed the sera with YU2 gp140-foldon coupled beads or blank control beads (Figure 1B). As expected, we found that, if the broad neutralizing activity of a particular serum could be adsorbed with YU2 gp120, it could also be adsorbed with YU2 gp140. However, for a subset of donors (#39, #21, and #15), the broad neutralizing activity of the sera was absorbed more efficiently with YU2 gp140 than YU2 gp120. This result may suggest that certain gp120 epitopes are better presented on the YU2 gp140-foldon trimer. Alternatively, a significant fraction of the serum neutralization breadth in these donors could be mediated by NAbs directed against gp41.

### Anti-MPER NAbs do not mediate a major fraction of serum neutralization breadth and potency in the donors studied

**Figure 2:**
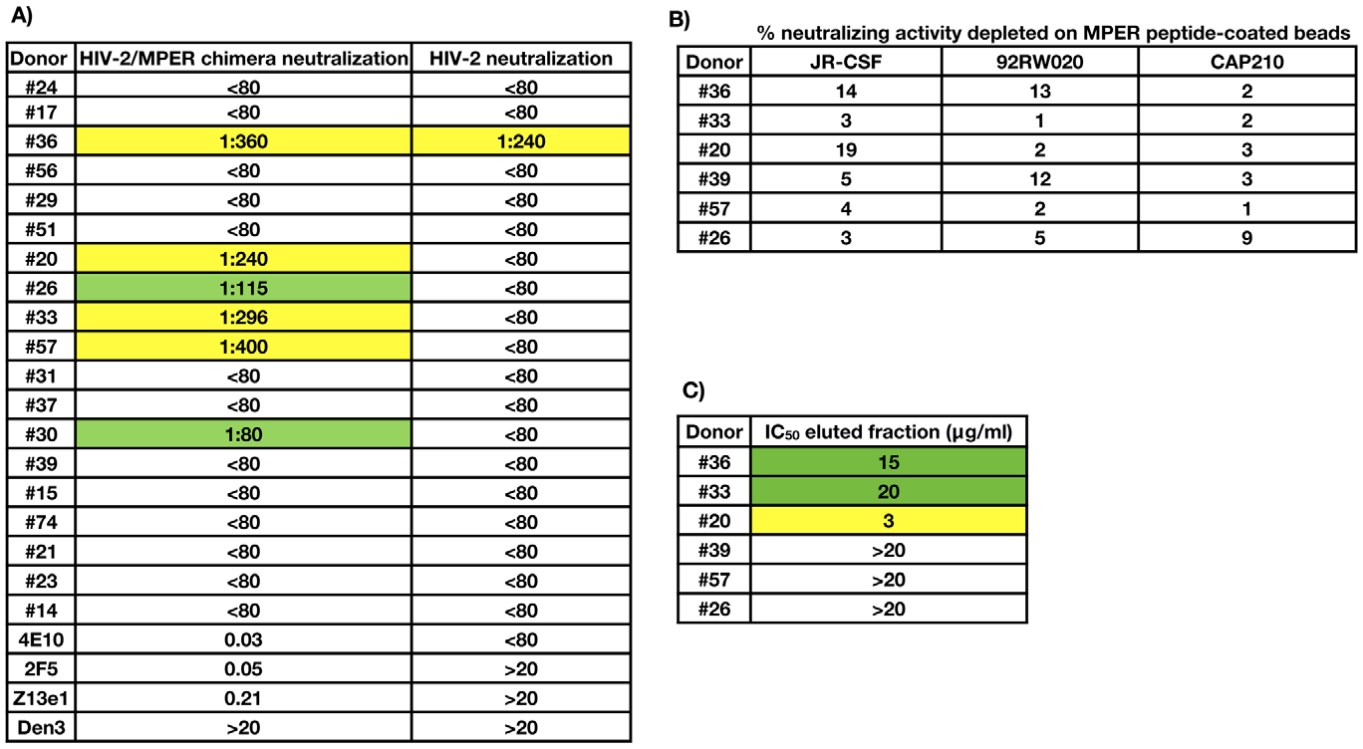
Contribution of MPER-directed NAbs to broad serum neutralization. A) Sera were tested for neutralizing activity against a chimeric HIV-2 virus containing the complete MPER region of gp41 [24]. Serum IC_50_ titers are expressed as 1/serum dilution. Boxes are color coded as follows: white squares, IC_50_≤1∶80; green squares, 1∶200>IC_50_>1∶80, yellow squares, 1∶500>IC_50_>1∶200. The IC_50_s of anti-MPER NAbs 2F5, 4E10, Z13e1 were also determined, expressed in µg/ml. The anti-Dengue antibody Den3 is included as a negative control. B) Serum neutralizing activity after adsorption with MPER peptide-coated beads. Sera were adsorbed with full-length MPER peptide-coated beads or blank control beads and then tested for neutralizing activity against a cross-clade pseudovirus panel, as described previously [54]. Percent serum neutralizing activity depleted on the MPER peptide was calculated using the equation = (1−(IC_50 blank beads_/IC_50 MPER beads_))*100. C) Neutralizing activity of serum antibodies eluted from the MPER-coated beads. Functional Abs were eluted from the MPER-coupled beads by exposing the beads to a series of increasingly acidic conditions as described [17]. ELISA assays were used to determine the concentration of total IgG in the eluted fraction. Boxes are color coded as follows: white squares, IC_50_>20 µg/ml; green squares, 20≥IC_50_>5 µg/ml, yellow squares, 5>IC_50_>1 µg/ml.

We next investigated the contribution of gp41-directed NAbs to broad serum neutralizing activity. Since the MPER region of gp41 contains the epitopes recognized by three broadly neutralizing monoclonal antibodies, 2F5, 4E10, and Z13e1, and is the only known neutralizing determinant on gp41, we focused on determining whether NAbs directed against this region were mediating broad serum neutralizing activity. As a first step, we tested the sera for neutralizing activity against a chimeric HIV-2 virus containing the complete MPER region of gp41 [24]. Based on this assay, six sera appeared to contain MPER-reactive NAbs (Figure 2A). One of these donors (#36) also neutralized the parental HIV-2 virus, indicating that this donor may be co-infected with HIV-2 or contain anti-HIV-1 NAbs that cross-react with HIV-2. Notably, since HIV-2/MPER chimeras are 1 to 2-logs more sensitive to NAbs 4E10 and Z13e1 than HIV-1 primary isolates [22], this assay may overestimate the contribution of anti-MPER antibodies to serum neutralization breadth and potency. Indeed, for all six of these donors, a substantial fraction of the broad serum neutralization could be adsorbed with monomeric gp120, demonstrating that anti-MPER NAbs probably do not dominate the overall serum neutralization breadth and potency. Nonetheless, to further investigate the contribution of anti-MPER NAbs to the serum neutralization breadth in these six donors, we adsorbed the sera with MPER-coupled beads or blank control beads, as described previously [23]. As above, the adsorbed sera were then tested for neutralizing activity against a cross-clade pseudovirus panel (Figure 2B). In addition, the MPER-specific antibodies were eluted off the beads and tested for neutralizing activity against the clade B isolate JR-CSF (Figure 2C). As expected, for all six donors, the neutralizing activity of the serum after adsorption with MPER peptide-coated beads or blank beads were comparable, indicating that anti-MPER NAbs do not mediate a major fraction of the serum neutralization breadth and potency. However, for three donors, weak to moderate neutralizing activity against JR-CSF was observed in the fraction eluted from the MPER peptide-coupled beads, suggesting the presence of MPER-directed NAbs at low concentrations and/or low neutralizing potency in these sera.

### CD4bs and CRbs-directed antibodies contribute to broad serum neutralization in approximately 25% of donors

Previous serum mapping studies have evaluated CD4bs and CRbs-directed neutralizing activity in sera by performing serum adsorptions with gp120 point mutants that fail to react with existing mAbs directed against these epitopes. However, a caveat to this approach is that some CD4bs or CRbs NAbs may be insensitive or only partially sensitive to these particular mutations. Notably, a recently reported broadly neutralizing CD4bs-directed NAb binds to the D368R gp120 variant, often considered a prototypic non-CD4bs Ab binding gp120, with higher affinity than the wild-type (WT) gp120 molecule [34]. Therefore, as an alternative to use of the D368R gp120 variant, we developed a serum adsorption method based on antibody competition. Using this method, serum adsorptions to Env are performed in the presence of saturating concentrations of a non-neutralizing competitor mAb (the competitor Ab must be non-neutralizing so its presence will not affect the results of the neutralization assay). In principle, Abs directed against epitopes overlapping that of the competitor Ab will fail to bind to the Env-coated beads. Since the non-neutralizing CD4bs-directed mAb b6 has been shown to compete with both CD4bs and CRbs-directed Abs for binding to gp120 [35], b6 was used as a competitor in these experiments.

To first validate this method, we adsorbed b12 (a CD4bs-directed bNAb) with YU2 gp140-coated beads in the presence or absence of saturating concentrations of b6 or blank control beads. Indeed, all of the b12 neutralizing activity could be adsorbed with YU2 gp140-coated beads, but none of neutralizing activity could be adsorbed when the assay was performed in the presence of saturating concentrations of b6 (Figure S3).

**Figure 3:**
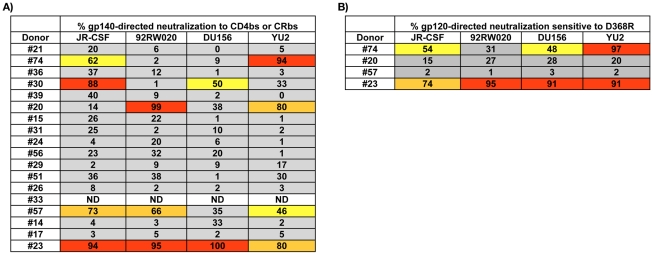
Analysis of CD4bs and CRbs-directed neutralizing activity. A) Sera were tested for neutralizing activity after adsorption with YU2 gp140-coupled beads in the presence or absence of saturating concentrations of mAb b6 or blank control beads. Percent gp140-directed neutralization to the CD4bs or CRbs was calculated using the equation = [(% neutralization absorbed by gp140 beads−% neutralization absorbed by gp140 beads in the presence of excess b6)/% neutralization absorbed by gp140 beads] *100. B) Sera were tested for neutralizing activity after adsorption with YU2 gp120 D368R coated beads or blank control beads. Percent gp120-directed neutralization sensitive to the D368R mutation was calculated using the equation = [(% neutralization absorbed by gp120 beads−% neutralization absorbed by gp120 D368R beads)/% neutralization absorbed by gp120 beads] *100. Boxes are color coded as follows: Gray, 0–45%, yellow, 45–65%; orange, 65–85%; red, 85–100%.

We next performed the assay using donor sera (Figure 3A). For one donor (#23), adsorption of the broad serum neutralizing activity with YU2 gp140 was completely inhibited by b6, suggesting that CD4bs or CRbs NAbs dominate the serum neutralization breadth and potency in this individual. For 4 additional donors, b6 inhibited 50–70% of broad serum neutralization indicating that CD4bs or CRbs Abs contribute significantly to the overall serum neutralization breadth and potency but not exclusively. For the remaining 12 donors, none or only a small fraction of the broad serum neutralization was blocked by b6, suggesting that CD4bs and CRbs-directed NAbs are of minor importance to the broad neutralizing responses in these individuals. A caveat to note is that anti-CD4bs or anti-CRbs NAbs that mediate the serum neutralization against some isolates (but not others) could be present in these 12 sera, but their epitopes may not be properly expressed in the context of YU2 gp140 used in the adsorption experiments.

Since the b6-blocking approach does not discriminate between CD4bs and CRbs-directed NAbs, we also performed serum adsorptions with a D368R gp120 variant that fails to bind CD4 and most, although not all, CD4bs-directed mAbs [25]. In two cases, a positive correlation was observed between the b6-inhibition adsorptions and the D368R adsorptions, indicating that the NAbs contributing to serum neutralization breadth in these donors are directed against the CD4bs (Figure 3B). However, for donors #20 and #57, the NAb specificities mediating serum neutralization breadth and potency competed with mAb b6 for gp140 binding yet did not exhibit sensitivity to the D368R mutation. It is possible that these NAbs are directed against the CRbs or novel epitopes that overlap the b6 epitope. Alternatively, these NAbs may be directed against the CD4bs but are insensitive to the D368R substitution.

### Dissecting the epitope fine specificities of serum NAbs using a large panel of mutant pseudoviruses

**Figure 4:**
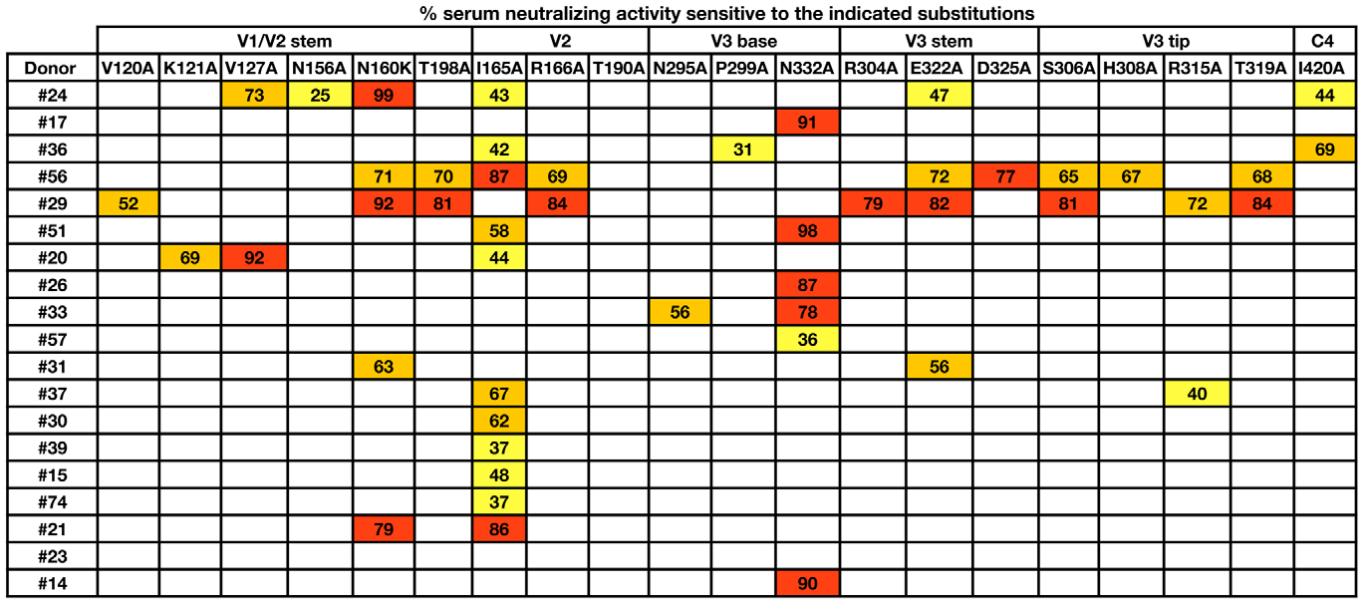
Effects of single amino acid substitutions on serum neutralizing activity against JR-CSF. Sera were tested for neutralizing activity against 97 JR-CSF pseudoviruses incorporating single amino acid substitutions. Percent serum neutralizing activity sensitive to the indicated substitution was calculated using the equation = (1−(IC_50 variant_/IC_50 WT_))*100. Boxes are color coded as follows: yellow, 20–50%; orange, 50–80%; red, 80–100%. The first and second rows designate the gp120 domain and the single amino acid substitution, respectively. JR-CSF pseudovirus variants that were similarly sensitive as wild type JR-CSF to all of the sera or variants that were globally sensitive to serum neutralization are not shown but are listed in Table S1.

The inability to adsorb a significant fraction of the broad serum neutralization with recombinant Env proteins in approximately one third of the donors prompted us to develop mapping strategies based on functional assays. As a first approach, we tested all of the sera for neutralizing activity against approximately 100 JR-CSF pseudoviruses incorporating single amino acid substitutions (Figure 4 and Table S1). In principle, if the serum neutralization against JR-CSF were mediated by a small number of NAb specificities, this activity would be diminished against pseudoviruses incorporating mutations that disrupt the epitopes targeted by these bNAbs. Indeed, for approximately 75% of the donors, potent serum neutralization against JR-CSF was abrogated by single amino acid substitutions. Notably, for the donor from whom PG9 and PG16 were isolated (#24), the N160K substitution in the context of JR-CSF Env resulted in complete viral escape from serum neutralization. Considering that this glycan is essential for PG9 and PG16 neutralizing activity [26], but does not affect the binding or neutralization profiles of any of the other Abs we tested (Table S2), this result suggests that this donor's potent serum neutralization against JR-CSF is entirely mediated by PG9, PG16, and similar antibodies. The N160K mutation also diminished serum neutralization against JR-CSF in four additional donors (#56, #29, #31, and #21), suggesting the presence of NAbs that target epitopes overlapping that of PG9 and PG16 in these sera. Alanine mutations in other regions of the V1/V2 and V3 loops also abrogated serum neutralizing activity against JR-CSF in these donors, further suggesting involvement of these regions in forming the epitopes recognized by these NAbs. Additionally, the broad and potent serum neutralizing activity in the five sera above could be not be efficiently adsorbed with monomeric gp120 or recombinantly trimerized gp140, indicating that these NAbs bind poorly to recombinant Env proteins. However, it is worth noting that a small number of gp120s have been identified that react weakly with PG9 [26], suggesting that it may be possible to adsorb these donor sera on certain gp120s.

In five different donors (#17, #51, #26, #14, #33), the N-linked glycan at position 332 at the base of the V3 loop of gp120 was critical for potent serum neutralization against JR-CSF. Since this glycan is also critical for 2G12 recognition [36], but does not significantly affect the neutralization profiles of other neutralizing mAbs we tested (Table S2), this result raised the possibility that the donor sera target epitopes overlapping that of 2G12. Interestingly, 2G12 also requires the glycan at position 295 for neutralizing activity, and one of the donors (#33) also exhibited sensitivity to this mutation. The glycan-dependent nature of the epitopes targeted by the NAbs in these sera is discussed below. Interestingly, a significant fraction of the broad serum neutralizing activity in all five of these sera could be adsorbed with monomeric gp120 and trimeric gp140, indicating that the epitopes targeted by these NAbs are expressed on recombinant forms of Env.

Approximately 21% of the donors exhibited significant sensitivity to the I165A mutation located in the V2 loop of gp120. To gain insight into the potential epitopes targeted by these NAbs, we tested a panel of neutralizing monoclonal antibodies for sensitivity to this mutation. Interestingly, only the trimer-specific NAbs 2909, 2.2G, and 2.3E required this residue for potent neutralizing activity (Table S2). Although these NAbs are strain-specific, they recognize quaternary epitopes involving the V2 and V3 loops of gp120 [37], [38]. For most of these donors, the broad neutralizing activity could not be adsorbed with monomeric gp120, suggesting that these NAbs may also target epitopes that are preferentially expressed on trimeric HIV-1 Env. Alternatively, the serum NAbs may target novel viral epitopes that are disrupted by the I165A mutation.

Of note, the contribution of CD4bs-directed NAbs could not be assessed using the mutant virus approach because pseudoviruses incorporating mutations in this region are non-infectious. Indeed, the donor sera identified above with CD4bs-directed neutralizing activity did not exhibit significant sensitivity to any of the pseudovirus mutants in our panel (Figure 4 and Table S1) consistent with the critical residues that form the epitopes recognized by these bNAb specificities being located in the CD4bs and the corresponding variants being absent from the pseudovirus panel. A second caveat of this assay is that certain mutations impart global sensitivity to serum neutralization, and therefore the effect of these residues in forming the epitopes recognized by the broadly neutralizing specificities in the sera could not be assessed. Interestingly, several of the substitutions that conferred a global neutralization sensitive phenotype were located in the V2 and V3 loops and the CRbs (Table S1), suggesting that these residues play a role in restricting antibody access to potentially neutralizing epitopes.

### A small number of antibody specificities mediate potent, cross-clade serum neutralizing activity in individual donors

**Figure 5:**
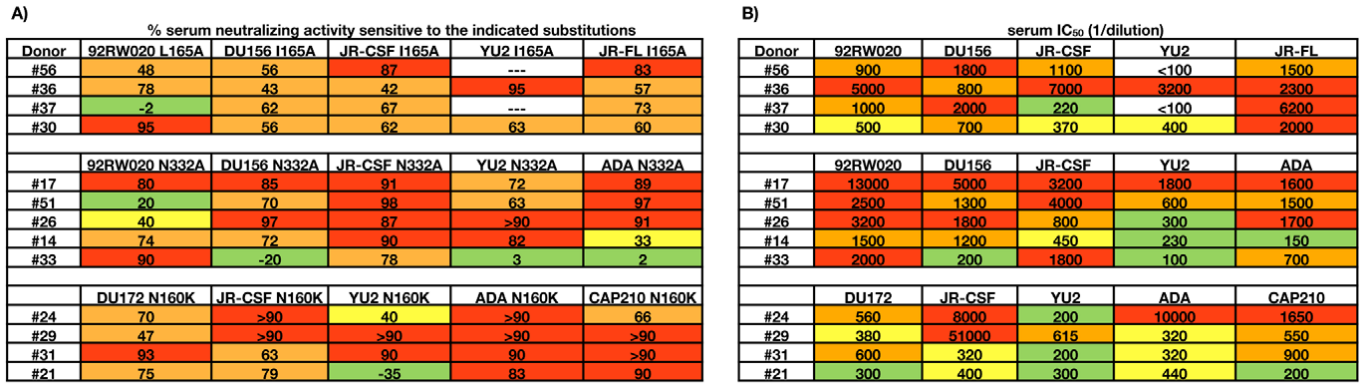
Effects of single amino acid substitutions on broad serum neutralizing activity. A) Sera were tested for neutralizing activity against a cross-clade pseudovirus panel incorporating single amino acid substitutions using a single round of replication pseudovirus assay and TZM-bl target cells, as described previously [54]. Percent serum neutralizing activity sensitive to the indicated substitution was calculated using the equation = (1−(IC_50 variant_/IC_50 WT_))*100. Boxes are color coded as follows: green, 0–20%; yellow, 20–40%; orange, 40–80%; red, 80–100%. (—) indicates that the isolate was neutralized by the serum at <1∶100 dilution. B) Serum neutralizing activity against the 5-pseudovirus panel. Serum IC_50_ titers are color coded as follows: white squares, IC50≤1∶100; green squares, 1∶300>IC50≥1∶100, yellow squares, 1∶500>IC50≥1∶300; orange squares, 1∶1500>IC50≥1∶500, red squares, IC50≥1∶1500.

We next investigated whether the NAb specificities that were mediating the potent serum neutralization against JR-CSF were also mediating the breadth of serum neutralization. Since most of the sera exhibited sensitivity to the N160K, N332A, or I165A mutations, we introduced these mutations into a cross-clade pseudovirus panel and then tested the corresponding sera for neutralization against these variants (Figure 5A). In the context of 92RW020 (clade A), the N160K mutation resulted in a loss of viral infectivity and was therefore excluded from the N160K panel. For all of the donors, the single amino acid substitution that diminished serum neutralization against JR-CSF also abrogated cross-clade serum neutralization, suggesting that the broad and potent serum neutralization is mediated by a limited number of antibody specificities. In a minority of cases, the serum neutralizing activity against a particular isolate was not affected by the single amino acid substitution. However, these isolates were usually not potently neutralized by the serum (Figure 5B). For example, for donor #24, the N160K substitution only reduced the serum neutralizing activity against YU2 by 40%, but the serum neutralizing titer against this isolate was at least 10-fold lower than most of the other viruses on the panel. Thus, although there may be several bNAb specificities in these sera, the most potent neutralizing activity is likely only mediated by a small subset of bNAbs.

### Analysis of the glycan-dependent epitopes targeted by the serum NAbs

**Figure 6:**
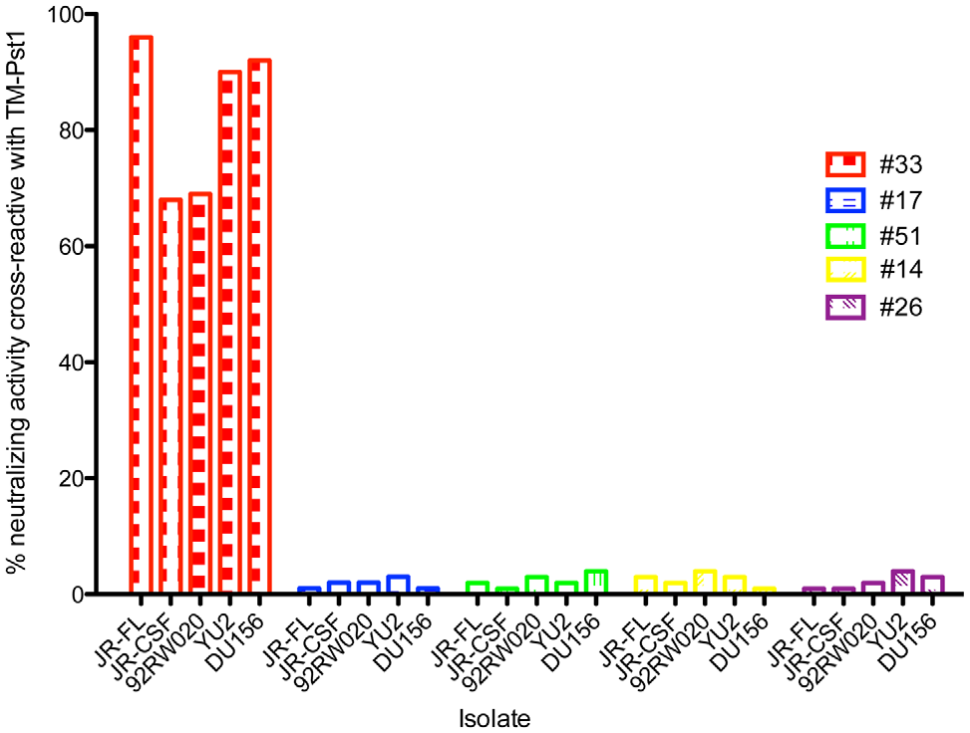
Analysis of glycan-specific activity in sera using TM-Pst1 coated beads. Sera were tested for neutralizing activity after adsorption with TM-Pst1-coupled beads or blank control beads. Neutralizing activity was assessed against a cross-clade pseudovirus panel using a single round of replication pseudovirus assay using TZM-bl target cells, as described [54]. Percent neutralizing activity cross-reactive with TM-Pst1 was calculated using the equation = (1−(IC_50 blank bead-adsorbed serum_/IC_50 Pst1-adsorbed serum_))*100.

We next sought to determine whether 2G12-like antibodies were mediating the serum neutralization breadth and potency in donors who exhibited sensitivity to the N332A mutation. Since 2G12 binding to gp120 is completely inhibited by 1M mannose [36], we first evaluated whether high concentrations of mannose could inhibit binding of the serum NAbs to YU2 gp140. For this, we performed serum adsorptions in the presence of 1M mannose or 1M glucose (negative control). As expected, 2G12 neutralizing activity was retained after adsorption with gp120-coupled beads in the presence of 1M mannose and depleted when the adsorption was performed in the presence of 1M glucose (Table S3). In contrast, the neutralization depletion efficiency of all five N332A-sensitive sera was similar in the presence of both mannose and glucose, indicating that the monosaccharide mannose does not inhibit the interaction of the NAbs in the sera with YU2 gp140 (Table S3). Notably, the crystal structure of 2G12 complexed with oligomannoses shows that 2G12 primarily recognizes the terminal mannose on the D1 arm of Man_9_GlcNAc_2_ [39]. If the sera contain glycan-specific NAbs that require several mannose residues for high affinity interaction, the monosaccharide mannose may not efficiently inhibit binding of these antibodies to gp120. Therefore, in a second approach we investigated whether the N332A-sensitive NAbs in the sera would react with the high-mannose glycans presented on a heavily N-glycosylated yeast protein, referred to as TM-Pst1, that has been shown to bind 2G12 with high affinity and inhibit 2G12 neutralization of HIV-1 pseudoviruses [40]. For these experiments, we performed serum adsorptions with TM-Pst1-coupled beads or blank control beads. Since all of the sera contained TM-Pst1 binding antibodies, ELISA assays were used to confirm depletion efficiency (data not shown). As above, we next tested the adsorbed sera for neutralizing activity against a cross-clade pseudovirus panel (Figure 6 and Figure S4). Interestingly, for the donor that exhibited sensitivity to mutations at positions 295 and 332 (#33), 70–95% of the serum neutralization was adsorbed with the TM-Pst1 protein. Furthermore, the antibodies eluted off the beads bound to gp120, displayed cross-clade neutralizing activity, and exhibited sensitivity to the N332A mutation (Figure S5). Thus, it appears likely that this donor's broad and potent serum neutralizing activity is mediated by bNAbs that target the oligomannose cluster on the HIV-1 glycan shield. In contrast, for the remaining four donors, the broad neutralizing serum activity could not be adsorbed with TM-Pst1-coupled beads. One possible explanation is that these NAbs bind to glycan epitopes distinct from the 2G12 epitope. Alternatively, these NAbs may bind to protein epitopes that are conformationally dependent on the glycan at position 332. To further examine whether any of these sera contain anti-glycan NAbs that bind to epitopes overlapping that of 2G12, we performed competition ELISA experiments using biotinylated mAb 2G12 (Figure S6). None of the sera, including the serum that could be adsorbed on the yeast TM-PstI protein, decreased the binding of biotinylated mAb 2G12 to JR-CSF gp120, suggesting that any glycan-specific NAbs in these sera bind to epitopes distinct from that of 2G12.

**Figure 7:**
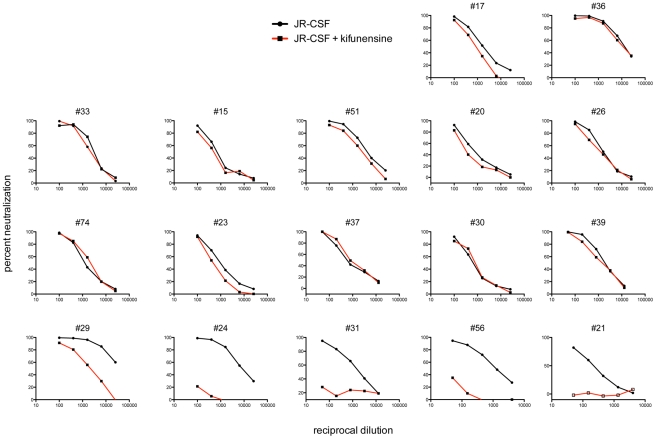
Serum neutralization activity against kifunensine-treated JR-CSF pseudoviruses. Sera were tested for neutralizing activity against JR-CSF pseudoviruses produced in the presence or absence of the glycosidase inhibitor kifunensine. Kifunensine-treated pseudoviruses were produced by treating 293T cells with 25 µM kifunensine on the day of transfection. Neutralizing activity was assessed using a single round of replication pseudovirus assay and TZM-bl target cells, as described previously [54].

**Figure 8:**
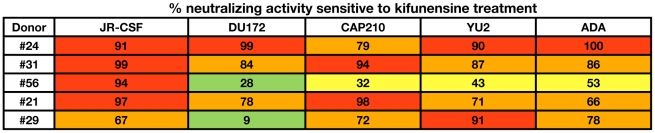
Effect of kifunensine treatment on broad serum neutralization. Sera were tested for neutralizing activity against a cross-clade panel of pseudoviruses produced in the presence or absence of the glycosidase inhibitor kifunensine. Kifunensine-treated pseudoviruses were produced by treating 293T cells with 25 µM kifunensine on the day of transfection. Neutralizing activity was assessed using a single round of replication pseudovirus assay and TZM-bl target cells, as described previously [54]. Percent neutralizing activity sensitive to kifunensine treatment was calculated using the equation = (1−(IC_50 kifunensine treated virus_/IC_50 WT virus_))*100. Boxes are color coded as follows: green, 0–30%; yellow, 30–60%; orange, 60–90%; red, 90–100%.

Next, we further assessed the contribution of PG9 and PG16-like antibodies to serum neutralization breadth and potency in donors who exhibited sensitivity to the N160K mutation. We have previously observed that pseudoviruses produced in cells that have been treated with kifunensine, a mannose analogue that inhibits type-1 endoplasmic reticulum (ER) and Golgi α-mannosidases, are resistant to PG9 and PG16 neutralization (Doores *et al.*, submitted). Surprisingly, we found that other NAbs, including those that bind to quaternary epitopes on trimeric Env, neutralized kifunensine-treated pseudoviruses with similar potency as wild-type (WT) pseudoviruses (Table S4). Therefore, to further investigate whether PG9 and PG16-antibodies mediate the potent serum neutralizing activity observed in donors who exhibit sensitivity to the N160K mutation, we tested these sera for neutralizing activity against JR-CSF pseudoviruses produced in the presence of kifunensine. For comparison, we also tested sera from donors whose serum neutralizing activity was unaffected by this mutation. Indeed, we found that only the five sera that exhibited sensitivity to the JR-CSF N160K mutation showed markedly diminished neutralizing activity against kifunensine-treated JR-CSF pseudoviruses (Figure 7). To determine whether the kifunensine-sensitive NAbs were also mediating broad serum neutralization, we next tested these five sera for neutralization against a cross-clade panel of pseudoviruses produced in the presence of kifunensine. For four out of the five donors, the broad serum neutralizing activity was almost completely abolished against kifunensine-treated pseudoviruses (Figure 8). Notably, for donor #29, both kifunensine treatment and the N160K mutation had only moderate effects on serum neutralization against the DU172 isolate, further suggesting a correlation between sensitivity to the N160K mutation and kifunensine treatment. Based on these results, it appears highly likely that PG9 and PG16-like antibodies mediate most of the broad and potent serum neutralizing activity in the four donors.

## Discussion

We have used several established and new approaches to map the serum NAb specificities in 19 donors, mostly infected with non-clade B viruses, who exhibit remarkably broad and potent serum neutralizing activity. This study extends our previous serum mapping studies, which only focused on a small number of patients with relatively limited serum neutralization breadth and potency [24]. Here, we found that the broad serum neutralizing activity in about one third of the donors in our cohort could not be efficiently adsorbed with recombinant monomeric gp120 or recombinantly trimerized gp140, suggesting the possible presence of bNAbs that target quaternary epitopes. Interestingly, in a subset of donors, the broad serum neutralizing activity was more efficiently adsorbed with recombinantly trimerized gp140 than monomeric gp120, indicating the presence of NAbs directed against gp120 epitopes that are somewhat better exposed on the YU2 gp140-foldon trimer or that the NAbs are directed against gp41. A caveat associated with these conclusions is that a single Env protein was used for the serum adsorption studies; it is possible that some of the broadly neutralizing specificities in these sera would react differently with Env proteins derived from other isolates.

MPER-directed neutralizing activity was detected in 30% of the donors, but these NAbs did not substantially contribute to the overall breadth and potency observed in the sera. Indeed, the broad and potent serum neutralizing activity for most donors in our cohort could either be adsorbed with monomeric gp120 and/or was abrogated by specific mutations located in gp120, further suggesting that gp41-directed NAb specificities do not mediate the overall serum neutralization breadth and potency in these donors. This result is in agreement with previous serum mapping studies that suggest NAbs directed against the MPER rarely mediate broad and potent serum neutralization [16], [17], [18], [22].

Previous serum mapping studies have shown that, in some individuals, NAbs directed against the CD4bs or CRbs mediate broad serum neutralization [16], [17], [25], [41], [42]. Furthermore, four broadly neutralizing CD4bs-directed mAbs have been isolated from HIV-1 infected donors [34], [43,Wu *et al.*, submitted]. To evaluate whether CD4bs or CRbs-directed NAbs were mediating the serum neutralization breadth and potency in these donors, we performed serum adsorptions in the presence or absence of saturating concentrations of the non-neutralizing CD4bs-directed mAb b6. We found that, in 5 out of the 17 donors, a significant proportion of the overall broad and/or potent serum neutralizing activity was mediated by receptor binding site-directed NAbs, and in one donor, nearly all of the broad serum neutralizing activity could be attributed to the b6-competed fraction. In two donors, similar results were obtained when a CD4bs-altered mutant (D368R) was used for adsorptions, indicating that the NAbs contributing to the serum neutralization breadth in these donors are directed against the CD4bs.

Although serum adsorption studies provide valuable insight into the epitopes targeted by NAbs that react with recombinant Env or linear peptides, defining the epitopes recognized by trimer-specific antibodies requires the use of functional assays. By testing the sera for neutralizing activity against a large panel of JR-CSF pseudovirus mutants incorporating single amino acid substitutions, we were able to define the epitopes recognized by the bNAb specificities in the context of the native trimer. Interestingly, we found that single amino acid mutations in Env frequently generated viruses that were far less sensitive to serum neutralization than the wild-type JR-CSF virus. Incorporation of these single substitutions into a cross-clade pseudovirus panel similarly generated viruses that were far more resistant to neutralization by certain sera than the corresponding wild-type viruses. These findings suggest that the broad and potent serum neutralizing activity in these donors is mediated by a limited number of antibody specificities. Notably, for the donor from whom PG9 and PG16 were isolated, the neutralization properties of the serum mirrored that of PG9 and PG16. These results complement the previous observation that PG9 and PG16 could recapitulate this donor's broad and potent serum neutralization against most isolates [26]. Thus, it appears highly likely that PG9, PG16, and related antibodies mediate this donor's serum neutralization breadth and potency. In the context of the single amino acid variant viruses, it should be noted that although it is most likely that the substitutions directly define residues involved in bNAb binding, it is also possible that the effects are due to transmitted effects from substitutions distant from the neutralizing epitope.

Interestingly, for 9 out of the 19 donors, the potent cross-clade serum neutralizing activity was mediated by NAbs dependent on specific glycans for epitope recognition. In four of these donors, the glycan at position 160 at the base of the V2 loop was critical for serum neutralization breadth and potency, suggesting the presence of NAbs that target epitopes overlapping that of PG9 and PG16. To evaluate the contribution of PG9 and PG16-like antibodies to broad and potent serum neutralizing activity, we used the following criteria: 1) the broad and potent serum neutralizing activity could not be efficiently adsorbed with recombinant Env proteins, 2) the N-linked glycan at position 160 was essential for serum neutralization breadth and potency, and 3) the broad and potent serum neutralizing activity was diminished against pseudoviruses produced in the presence of the glycan processing inhibitor kifunensine. Based on these criteria, PG9 and PG16-like NAbs were identified in approximately 21% of the donors we studied, demonstrating that this specificity is relatively common in donors that develop broad and potent serum neutralization.

The I165A substitution located in the V2 loop of gp120 abrogated broad serum neutralizing activity in 4 out of the 19 donors studied. Given that the trimer-specific NAbs 2909, 2.2G, and 2.3E require this amino acid for potent neutralizing activity, and that the broad serum neutralizing activity in these 4 donors could not be adsorbed with monomeric gp120, it appears highly likely that these bNAb specificities also target epitopes preferentially expressed on trimeric HIV-1 Env. Indeed, this epitope may be linked to the epitope recognized by PG9 and PG16.

We found that the N-linked glycan at position 332 was critical for broad and potent serum neutralizing activity in approximately 25% of donors. Considering that this N-linked glycan is also important for formation of the 2G12 epitope, we investigated whether 2G12-like antibodies were mediating the serum neutralization breadth and potency in any of these donors. Notably, the presence of 2G12-like antibodies in HIV-1 positive sera has also been discussed recently by others [22], [44]. In one donor, a large fraction of the broad serum neutralizing activity cross-reacted with a yeast glycoprotein that expresses homogenous Man_8_GlcNac_2_ carbohydrates and binds 2G12 with high affinity, suggesting the presence of bNAbs that bind directly to the glycan shield. This donor's serum neutralizing activity was also dependent on the presence of the N-linked glycan at position 295, a second N-linked glycan required for 2G12 recognition [36]. Interestingly, the sera from this individual did not inhibit 2G12 binding to gp120, suggesting that this bNAb specificity may bind to a glycan epitope distinct from that of 2G12. Considering that protein-carbohydrate interactions are typically weak, perhaps this antibody specificity gains the necessary avidity by cross-linking two protomers within a trimer [45], [46]. Hence, low affinity binding to monomeric gp120 may also explain the lack of competition with 2G12. Another observation worth noting is that only a single individual in our study was found to have bNAb specificities targeting the glycan shield, indicating that these types of antibodies rarely mediate serum neutralization breadth and potency. However, it has recently been shown that 2G12 is unusually efficient in protection relative to its neutralizing ability [9], suggesting that the glycan shield may have advantages as a vaccine target. Thus, it will be of interest to isolate the glycan-specific NAbs from this donor and test their protective efficacy to determine whether this is a general property of NAbs directed against the glycan shield. For the remaining four donors that exhibited N332A sensitivity, adsorption with TM-Pst1 coupled beads had no effect on the neutralizing activity of the sera; it therefore appears likely that the NAbs mediating serum neutralization breadth and potency in these donors bind to protein epitopes that are conformationally dependent on the glycan at position 332.

The observation that only a limited number of antibody specificities mediate serum neutralization breadth and potency in these donors contrasts with the results of a previous study by Scheid et. al in which no single broadly neutralizing monoclonal antibodies were isolated using single B cell sorting [27]. The authors of that study concluded that the serum neutralization breadth in their donors was due to the combined activity of a large number of antibody specificities that individually display limited breadth and potency. Indeed, this may be the case for certain donors, particularly those with relatively weak serum neutralization breadth and potency. However, in donors with extraordinarily potent serum neutralization, it appears that this activity is usually only associated with one or a few different specificities. Of note, serum neutralization breadth found to be mediated by a single specificity may in fact require multiple different NAbs circulating in the plasma that recognize overlapping targets within the same region. For example, a number of different CD4bs-directed NAbs may mediate broad serum neutralization in donors where all of the serum neutralizing activity can be mapped to this region.

**Figure 9:**
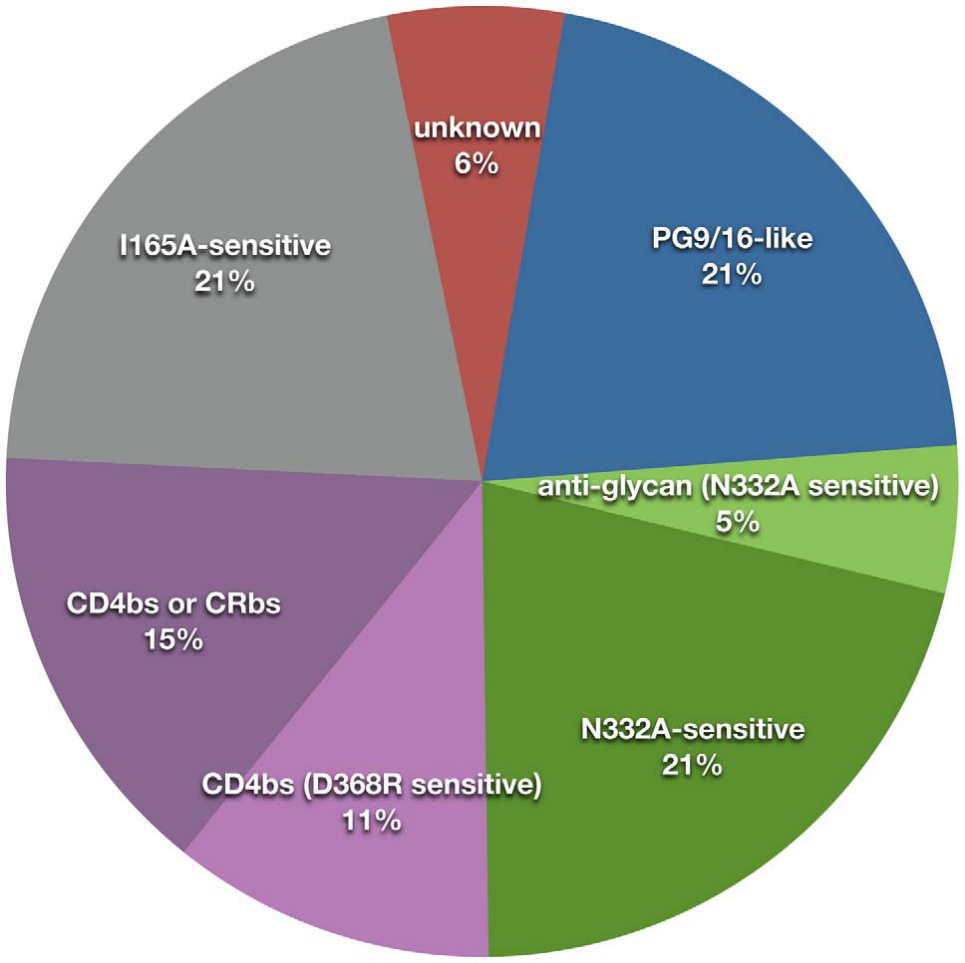
Summary of the predominant NAb specificities that mediate serum neutralization breath and potency across all donors. Percentages were calculated using only the predominant NAb specificity identified in each donor. The “unknown” category includes donors for which no predominant specificity was identified.

In summary, the data presented here show that the unusually potent cross-clade serum neutralizing activity observed in a selection of donors is mediated by a small number of antibody specificities that target conserved regions of Env to a significant extent (Figure S7 and Figure 9). Antibodies dependent on specific glycans for Env recognition were found to be responsible for this activity in approximately half of the donors we studied; 21% of these donors targeted epitopes overlapping those of PG9 and PG16 and 25% targeted epitopes that were dependent on the presence of an N-linked glycan at position 332. CD4bs and/or CRbs-directed NAbs contributed to serum neutralization breadth in 25% of donors, NAbs sensitive to the I165A substitution were identified in 21% of donors, and MPER-directed NAbs were present in a subset of donors but did not make a substantial contribution to the overall serum neutralization breadth and potency. Future studies, aimed at isolating and characterizing broadly neutralizing monoclonal antibodies from these donors, will be important for the molecular definition of the broadly neutralizing epitopes recognized by the donors.

## Materials and Methods

### Ethics statement

After obtaining written informed consent, sera and plasma were collected from HIV-1 infected volunteers in Rwanda, Zambia, Ivory Coast, Thailand, Kenya, Uganda, United Kingdom, and the United States. Eligible participants were age 18 or older, were HIV-1 infected for at least 3 years prior to the day of screening, were clinically asymptomatic, without evidence of progression to AIDS based on WHO Stage III or IV criteria or CD4 count <200 cells/mm3 and were not on antiretroviral therapy (ART) for at least 1 year. The study was reviewed and approved by the Republic of Rwanda National Ethics Committee; Emory University Institutional Review Board; University of Zambia Research Ethics Committee; Charing Cross Research Ethics Committee; UVRI Science and Ethics Committee; University of New South Wales Research Ethics Committee; and St. Vincent's Hospital and Eastern Sydney Area Health Service; Kenyatta National Hospital Ethics and Research Committee; University of Cape Town Research Ethics Committee; International Institutional Review Board, Mahidol University Ethics Committee; Walter Reed Army Institute of Research (WRAIR) Institutional Review Board; Ivory Coast Comité National d'Ethique des Sciences de la Vie et de la Santé (CNESVS).

### Human plasma samples

Sera and plasma were collected from a cohort of HIV-1 infected individuals (IAVI Protocol G), as previously described [15]. Samples were heat-inactivated at 55 °C for 1 h prior to use in neutralization assays.

### Antibodies

The panel of MAbs directed to HIV-1 Env included MAb b12, b13, and 15e, directed to epitopes overlapping the CD4bs of gp120 [43], [47]; 2G12, directed to a cluster of oligomannose residues on gp120 [36], [48]; A32, directed against an epitope comprised of the C1/C2/C4/and CD4i domains [49]; C11, directed against the C1 domain; F425/b4e8, directed to the V3 loop on gp120 [50]; ×5, directed to the CRbs binding site of gp120 [51]; PG9, PG16, 2.2G, 2.3E, and 2909, directed to quaternary epitopes comprised of the V2 and V3 loops of gp120 [26], [37], [38]; and 2F5, 4E10, and Z13e1, directed to the gp41 MPER [48], [52], [53].

### Pseudovirus production and neutralization assays

Pseudoviruses incorporating single alanine substitutions were generated by transfection of 293T cells with an Env-expressing plasmid and an Env-deficient genomic backbone plasmid (pSG3ΔEnv), as described previously [54]. Pseudoviruses were harvested 72 hours post transfection for use in neutralization assays. Neutralizing activity was assessed using a single round of replication pseudovirus assay and TZM-bl target cells, as described previously [54]. A chimeric HIV-2 clone containing the MPER of HIV-1 was derived from the parental HIV-2 7312A clone in which the HIV-2 Env MPER sequence QKLNSWDVFGNWFDLASWVKYIQ was replaced by the HIV-1 MPER sequence LALDKWASLWNWFDITKWLWYIK, as described [22]. To determine IC_50_ values, serial dilutions of plasma or mAb were incubated with virus and the dose-response curves were fitted using nonlinear regression. Serum antibody concentrations and antigen-binding activity were determined by ELISA, as described below.

### Production of monomeric and trimeric envelope glycoproteins

The envelope glycoproteins were expressed by transfecting the 293T cell line in serum-free medium (Invitrogen, Carlsbad, CA). In brief, the 293T cells were seeded in T225 flasks at a density of 1×10^5^ cells/cm^2^ and transfected with the expression plasmid YU2gp120/pcDNA3.1(−) for monomeric gp120 or YU2gp140-fibritin/pcDNA3.1(−) DNA for trimeric gp140 proteins. In addition, YU2 gp140-fibritin mutants were codon optimized for mammalian expression and synthesized (GeneArt AG, Germany). The D368R YU2 gp120 mutant was generated by Quik change mutagenesis (Stratagene). Four days post-transfection, cell culture supernatants were collected, clarified, filtered and two protease inhibitor tablets (Roche) per liter of supernatant were added to limit proteolysis. The gp120 or gp140-containing supernatants were stored at 4°C prior to purification. The supernatants containing the gp120 or gp140 proteins were applied to columns containing 10 ml of Galanthus nivalis lectin-bound agarose (Vector Laboratories). The column was then washed sequentially with 10 column volumes of phosphate-buffered saline (PBS) (pH 7.4) containing 0.5 M NaCl, followed by 10 column volumes of PBS (pH 7.4). The lectin-bound glycoproteins were eluted with a total of 12 column volumes of elution buffer (PBS buffer [pH 7.4] with 0.5 M methyl-D-mannopyranoside). The mannoside-eluted glycoproteins were pooled and the protein eluates were dialyzed against phosphate-buffered saline (PBS), pH 7.4, and concentrated with Amicon Ultra 30,000 MWCO centrifugal filter devices (Millipore, Bedford, MA). The purified proteins were subjected to sodium dodecyl sulfate-polyacrylamide gel electrophoresis and ELISA analysis, and protein purity was verified to approach 95% homogeneity.

### Serum adsorptions

Serum adsorptions with antigen-coupled beads were performed using tosyl-activated magnetic beads, as described previously [25]. 0.5 mg of gp120 and gp140 and 2 mg of TM-Pst1 were used for bead coupling. Two to three rounds of adsorption were performed to ensure complete removal of antigen-specific antibodies. Functional Abs were eluted from beads by exposing the beads to series of increasingly acidic conditions, as described [17]. For serum adsorptions performed in the presence of b6, gp140-coupled beads were pre-incubated with 200 µg/ml IgG b6 for 1 h at room temperature before adding serum. ). For adsorptions performed in the presence of monosaccharides, sera and mAb 2G12 were pre-incubated with 1M mannose or 1M glucose for 1 h at room temperature before adding to gp140 or gp120-coupled beads. Serum antibody concentrations and antigen-binding activity were determined by ELISA, as described below.

### ELISA assays

Ninety-six-well ELISA plates were coated overnight at 4 °C with 50 uL PBS containing 50 ng of goat anti-human IgG Fc (Pierce) or 100 ng gp120, gp140, or TM-Pst1 per well. The wells were washed four times with PBS containing 0.05% Tween 20 and blocked with 3% BSA at room temperature for 1 h. Serial dilutions of sera or mAb were then added to the wells, and the plates were incubated at room temperature for 1 hour. After washing four times, goat anti-human IgG F(ab')2 conjugated to alkaline phosphatase (Pierce), diluted 1∶1000 in PBS containing 1% BSA and 0.025% Tween 20, was added to the wells. The plate was incubated at room temperature for 1 h, washed four times, and the plate was developed by adding 50 uL of alkaline phosphatase substrate (Sigma) to 5 mL alkaline phosphatase staining buffer (pH 9.8), according to the manufacturer's instructions. The optical density at 405 nm was read on a microplate reader (Molecular Devices). Antibody concentration was calculated by linear regression using a standard concentration curve of purified IgG protein. Competition ELISAs were performed by pre-incubating 5-fold serum dilutions (starting at a 1∶25) on JR-CSF gp120-coated ELISA wells for 30 min at room temperature and then adding a concentration of biotinylated 2G12 previously determined to give a half-maximal binding signal. Biotinylated 2G12 was detected using alkaline phosphatase- conjugated streptavidin (1∶200 in dilution buffer; Pierce), and the plates were developed as described above. Endpoint titers of the plasma antibodies were defined as the last reciprocal serum dilution at which the OD signal was greater than twofold over the background signal.

## Supporting Information

Figure S1 Serum neutralization profiles of selected donors against a cross-clade pseudovirus panel. Sera were tested for neutralization activity against a cross-clade pseudovirus panel using a single round of replication pseudovirus assay and U87 target cells, as described previously [15]. Neutralization assays were performed using four-point serum dilutions (starting at 1∶100), and the values reported represent the highest dilution at which greater than or equal to 50% neutralization was achieved [15]. Neutralization scores have been assigned previously [15]. Donors with neutralization scores ≥2.5 have been designated as elite neutralizers, which represent the top 1% of all samples screened in the previous study. The presumed clade indicates the most predominate subtype or circulating recombinant form currently found within the cohorts from which the samples were collected.
(0.68 MB TIF)

Figure S2 Recognition of YU2 gp120 by the flow-throughs from YU2 gp120 and blank bead adsorptions. Bars indicate the relative end-point antibody ELISA titers in the plasma following mixing of the sera with beads coated with YU2 gp120 or blank beads.
(0.33 MB TIF)

Figure S3 Antibody b6 inhibits binding of b12 to gp140-coated beads. The bNAb b12 was tested for neutralizing activity after adsorption with YU2 gp140-coated beads in the presence or absence of saturating concentrations of b6 or blank control beads. Neutralizing activity was assessed using a single round of replication pseudovirus assay and TZM-bl target cells, as described [54].
(0.15 MB TIF)

Figure S4 TM-Pst1 reactive NAbs mediate serum neutralization breadth and potency in one donor (#33). Sera were tested for neutralizing activity after adsorption with TM-Pst1-coupled beads or blank control beads. Neutralizing activity was assessed using a single round of replication pseudovirus assay using TZM-bl target cells, as described [54].
(0.49 MB TIF)

Figure S5 Characterization of TM-Pst1 reactive NAbs from donor #33. Functional Abs were eluted from the TM-Pst1 coupled beads by exposing the beads to a series of increasingly acidic conditions as described [17]. ELISA assays were used to determine the concentration of total IgG in the eluted fraction. A) Binding of the TM-Pst1 eluted Abs to JR-CSF gp120, as determined by ELISA. B) Neutralizing activity of the TM-Pst1 eluted fraction. The TM-Pst1 eluted fraction neutralized JR-CSF (clade B) and 92RW020 (clade A) and showed reduced neutralizing activity against JR-CSF N332A.
(0.25 MB TIF)

Figure S6 Competition of N332A-sensitive sera with 2G12 for binding to gp120. Serum dilutions were added to JR-CSF gp120-coated ELISA wells and pre-incubated for 30 min prior to adding a concentration of 2G12 previously determined to give a half-maximal binding signal. Sera from HIV-negative donors were included as negative controls.
(0.15 MB TIF)

Figure S7 Summary of NAb specificities that mediate serum neutralization breadth and potency in individual donors. Colors and symbols indicate the percentage of overall serum neutralization breadth and potency assigned to the indicated specificity: gray (−), 0–25%; yellow (+), 25–50%; orange (++), 50–75%; red (+++), 75–100%. Percentages represent an average of all the isolates tested.
(0.24 MB TIF)

Table S1 Panel of JR-CSF pseudovirus variants. Sera were tested for neutralizing activity against JR-CSF pseudovirus variants incorporating single amino acid substitutions. Substitutions that resulted in global sensitivity to serum neutralization are highlighted in green.
(0.30 MB TIF)

Table S2 Neutralizing and binding activity of MAbs to gp120 and pseudovirus variants relative to WT. ^a^MAbs were tested for binding and neutralizing activity against gp120 and pseudovirus variants, respectively. A) All NAbs were tested against HIV-1 JR-CSF, except for 2909, 2.2G, and 2.3E, which were tested against HIV-1 SF162. B) ELISA binding assays were performed using gp120 captured from viral lysates, as described [50].^c^ Binding and neutralizing activity is reported as fold increase in IC_50_ value relative to WT and was calculated using the equation (IC_50_ variant/IC_50_ WT). Boxes are color coded as follows: gray, 0–10 fold IC_50_ increase; yellow, 10–100 fold IC_50_ increase; red, >100 fold IC_50_ increase.
(0.31 MB TIF)

Table S3 Ability of mannose to inhibit gp140 depletion of serum neutralizing activity. Sera were tested for neutralizing activity after adsorption with gp140-coupled beads in the presence of 1M mannose or 1M glucose (control). The glycan-specific bNAb 2G12 was included as a positive control. % increase in IC_50_ was calculated using the equation = (1−(IC_50_ blank beads/IC_50_ antigen-coated beads))*100. Boxes are color coded as follows: Green, 0–45%, yellow, 45–65%; orange, 65–85%; red, 85–100%.
(0.18 MB TIF)

Table S4 Effect of kifunensine treatment on neutralization by NAbs. NAbs were tested for neutralizing activity against pseudoviruses treated with kifunensine. Neutralizing activity is reported as fold increase in IC_50_ value relative to WT and was calculated using the equation (IC_50_ variant/IC_50_ WT). All mAbs were tested against HIV-1 JR-CSF, except for 2909, 2.2G, and 2.3E, which were tested against HIV-1 SF162. Boxes are color coded as follows: gray, 0–10 fold IC_50_ increase; yellow, 10–100 fold IC_50_ increase; red, >100 fold IC_50_ increase.
(0.14 MB TIF)
