# Supplementary figures and images for: A Limited Number of Antibody Specificities Mediate Broad and Potent Serum Neutralization in Selected HIV-1 Infected Individuals
Source: PLoS Pathog. 2010 Aug 5;6(8):e1001028. doi: 10.1371/journal.ppat.1001028 (PMC2916884; doi:10.1371/journal.ppat.1001028)

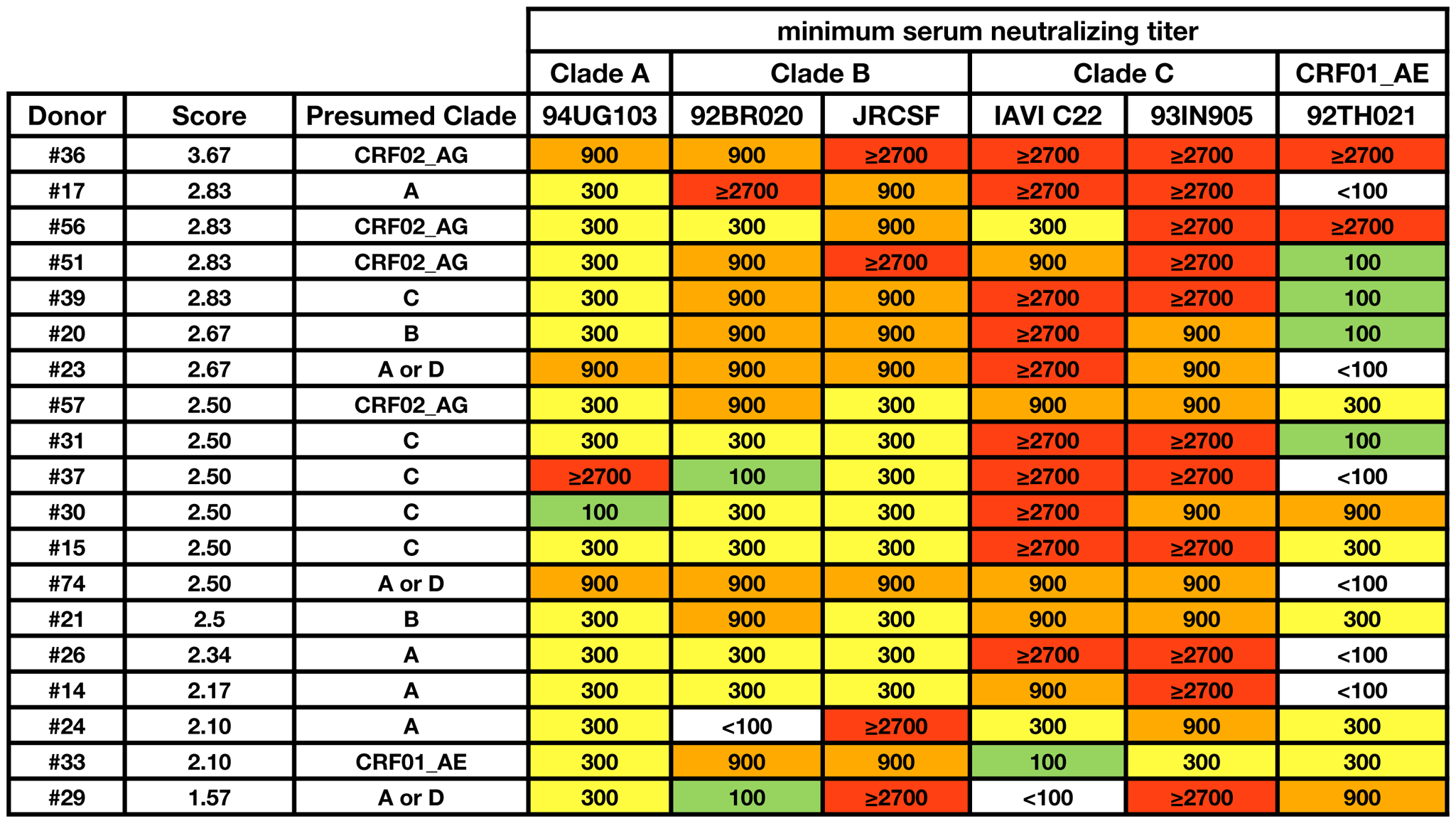

Supplement: Figure S1 — Serum neutralization profiles of selected donors against a cross-clade pseudovirus panel. Sera were tested for neutralization activity against a cross-clade pseudovirus panel using a single round of replication pseudovirus assay and U87 target cells, as described previously [15]. Neutralization assays were performed using four-point serum dilutions (starting at 1∶100), and the values reported represent the highest dilution at which greater than or equal to 50% neutralization was achieved [15]. Neutralization scores have been assigned previously [15]. Donors with neutralization scores ≥2.5 have been designated as elite neutralizers, which represent the top 1% of all samples screened in the previous study. The presumed clade indicates the most predominate subtype or circulating recombinant form currently found within the cohorts from which the samples were collected. (0.68 MB TIF) [file ppat.1001028.s001.tif]

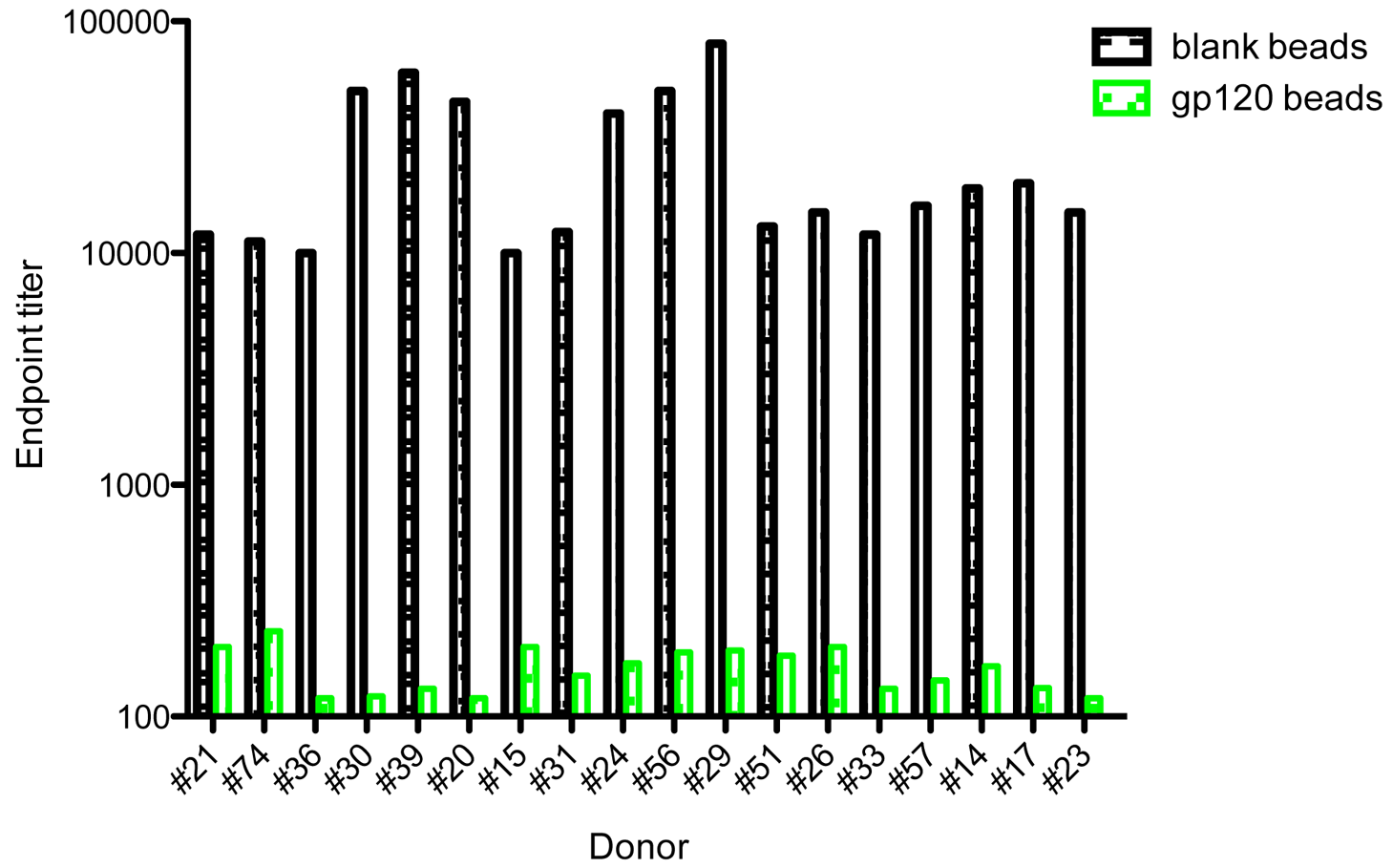

Supplement: Figure S2 — Recognition of YU2 gp120 by the flow-throughs from YU2 gp120 and blank bead adsorptions. Bars indicate the relative end-point antibody ELISA titers in the plasma following mixing of the sera with beads coated with YU2 gp120 or blank beads. (0.33 MB TIF) [file ppat.1001028.s002.tif]

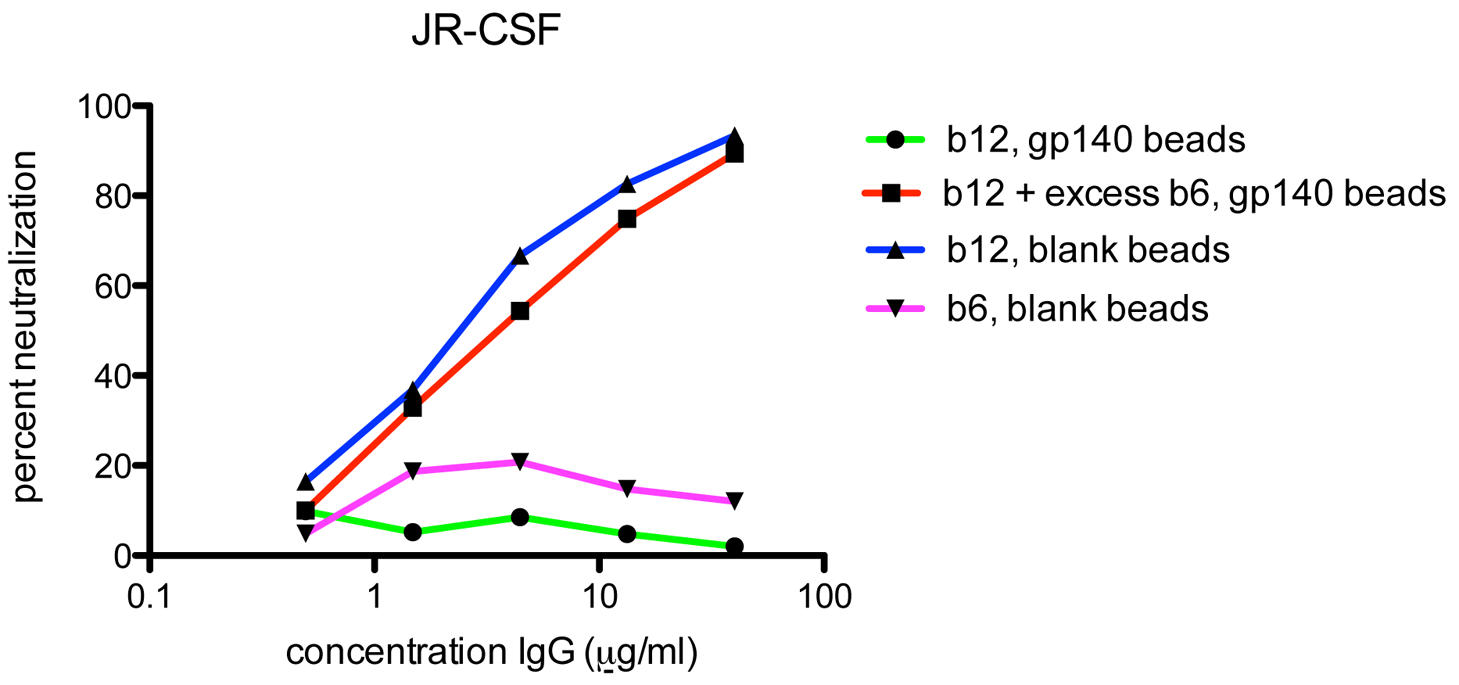

Supplement: Figure S3 — Antibody b6 inhibits binding of b12 to gp140-coated beads. The bNAb b12 was tested for neutralizing activity after adsorption with YU2 gp140-coated beads in the presence or absence of saturating concentrations of b6 or blank control beads. Neutralizing activity was assessed using a single round of replication pseudovirus assay and TZM-bl target cells, as described [54]. (0.15 MB TIF) [file ppat.1001028.s003.tif]

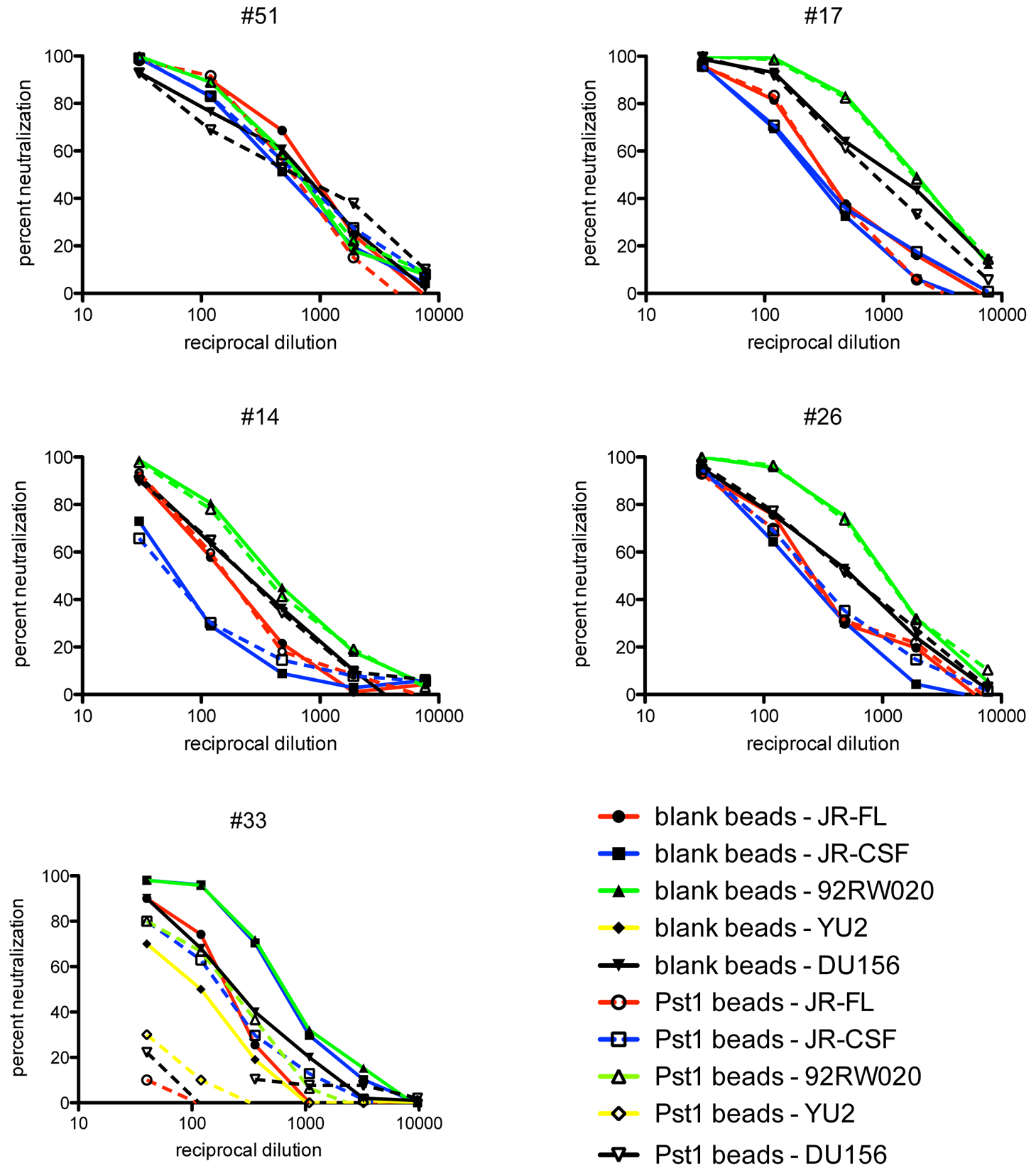

Supplement: Figure S4 — TM-Pst1 reactive NAbs mediate serum neutralization breadth and potency in one donor (#33). Sera were tested for neutralizing activity after adsorption with TM-Pst1-coupled beads or blank control beads. Neutralizing activity was assessed using a single round of replication pseudovirus assay using TZM-bl target cells, as described [54]. (0.49 MB TIF) [file ppat.1001028.s004.tif]

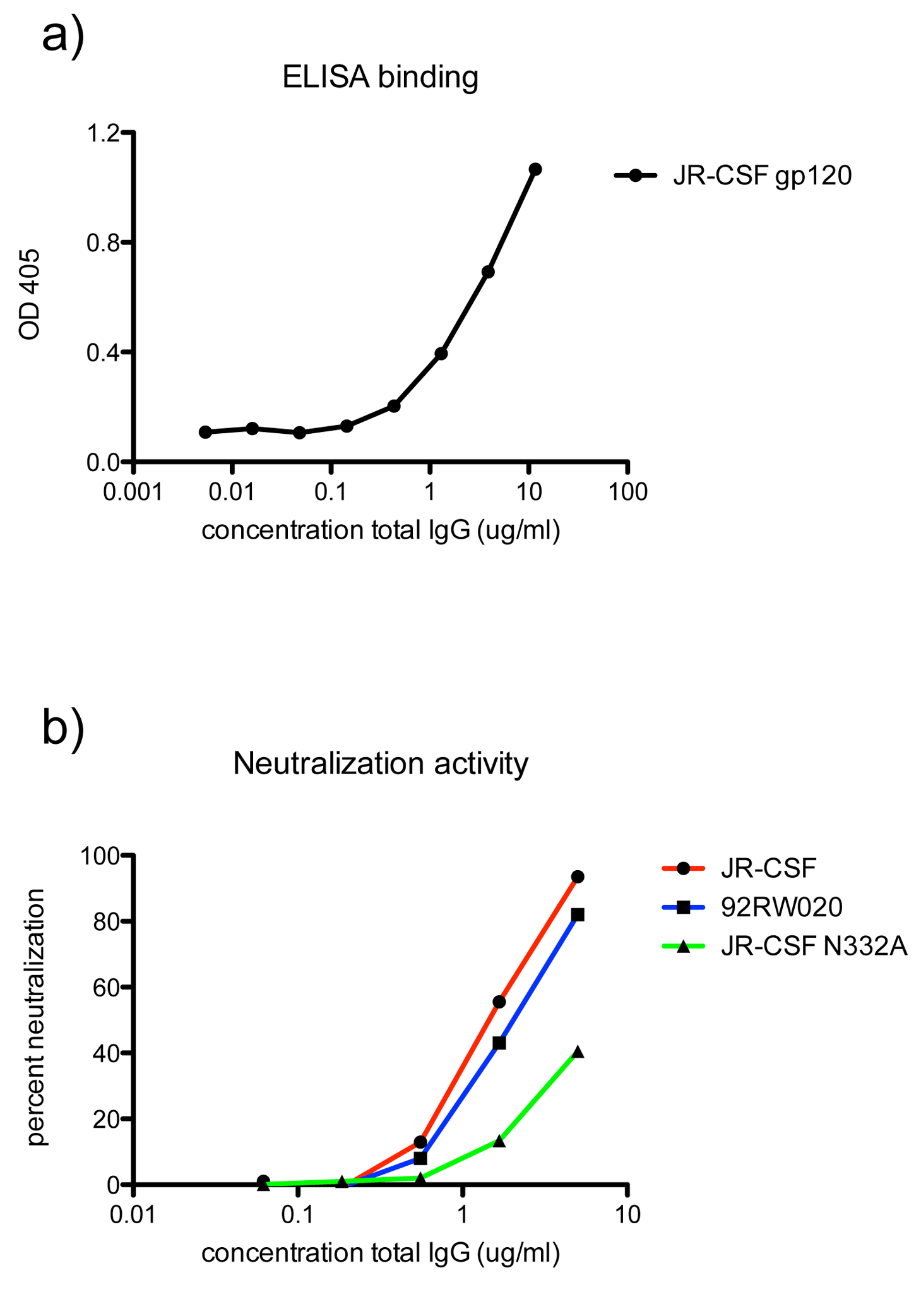

Supplement: Figure S5 — Characterization of TM-Pst1 reactive NAbs from donor #33. Functional Abs were eluted from the TM-Pst1 coupled beads by exposing the beads to a series of increasingly acidic conditions as described [17]. ELISA assays were used to determine the concentration of total IgG in the eluted fraction. A) Binding of the TM-Pst1 eluted Abs to JR-CSF gp120, as determined by ELISA. B) Neutralizing activity of the TM-Pst1 eluted fraction. The TM-Pst1 eluted fraction neutralized JR-CSF (clade B) and 92RW020 (clade A) and showed reduced neutralizing activity against JR-CSF N332A. (0.25 MB TIF) [file ppat.1001028.s005.tif]

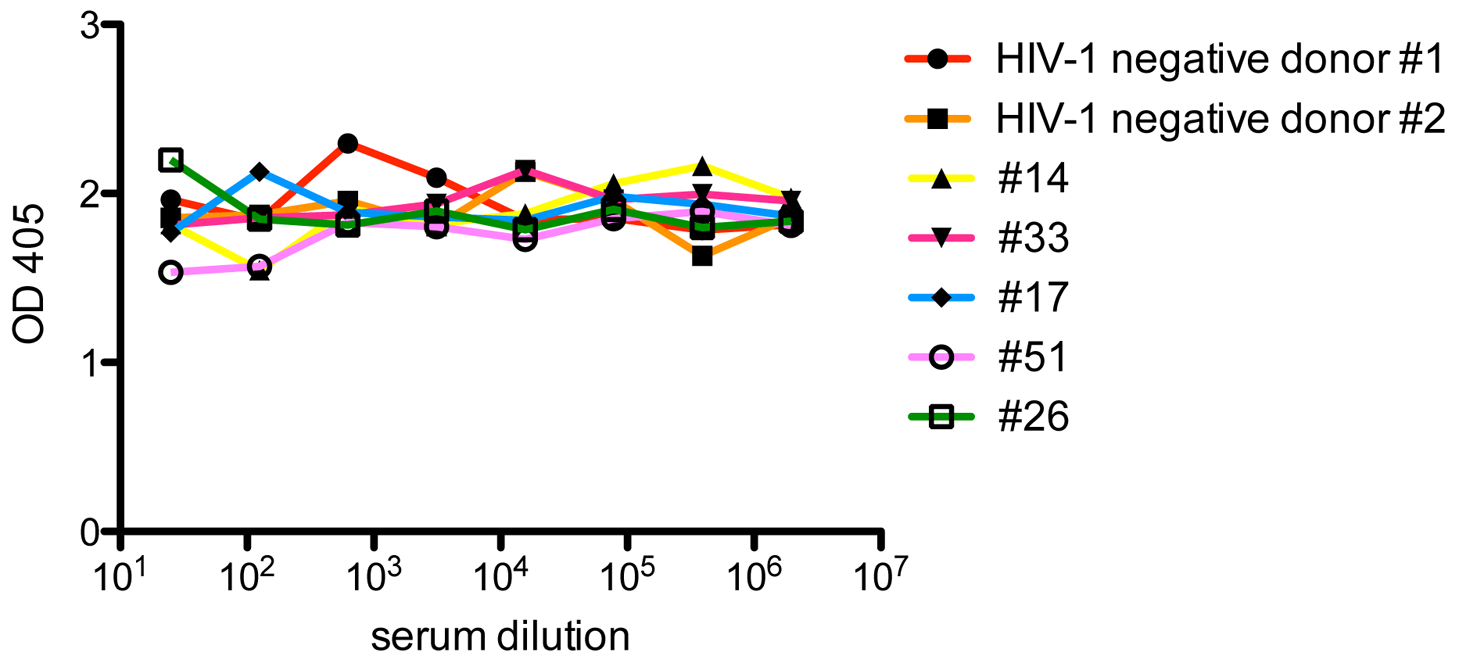

Supplement: Figure S6 — Competition of N332A-sensitive sera with 2G12 for binding to gp120. Serum dilutions were added to JR-CSF gp120-coated ELISA wells and pre-incubated for 30 min prior to adding a concentration of 2G12 previously determined to give a half-maximal binding signal. Sera from HIV-negative donors were included as negative controls. (0.15 MB TIF) [file ppat.1001028.s006.tif]

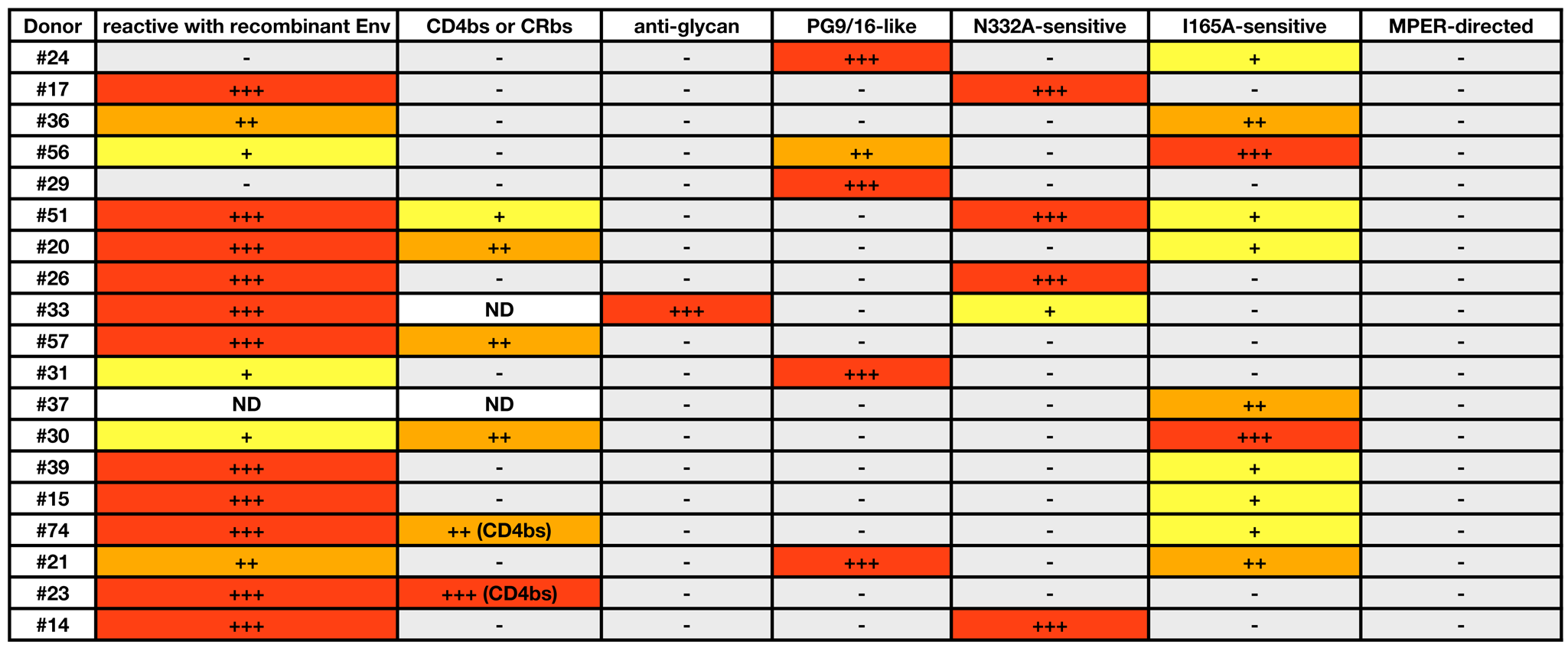

Supplement: Figure S7 — Summary of NAb specificities that mediate serum neutralization breadth and potency in individual donors. Colors and symbols indicate the percentage of overall serum neutralization breadth and potency assigned to the indicated specificity: gray (−), 0–25%; yellow (+), 25–50%; orange (++), 50–75%; red (+++), 75–100%. Percentages represent an average of all the isolates tested. (0.24 MB TIF) [file ppat.1001028.s007.tif]

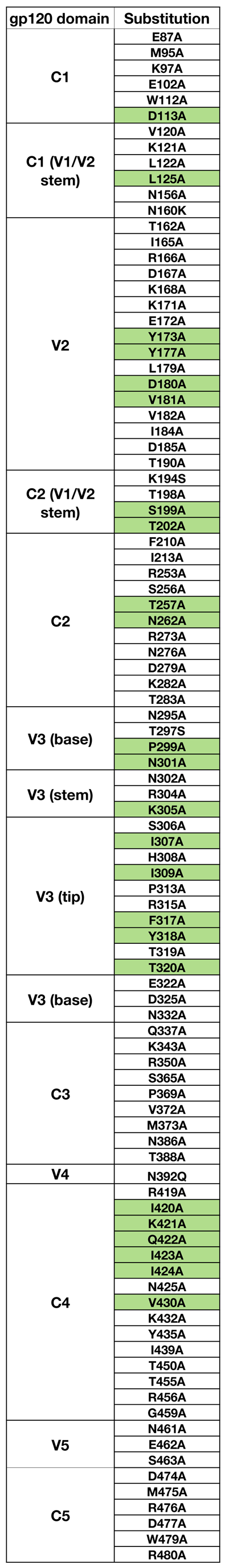

Supplement: Table S1 — Panel of JR-CSF pseudovirus variants. Sera were tested for neutralizing activity against JR-CSF pseudovirus variants incorporating single amino acid substitutions. Substitutions that resulted in global sensitivity to serum neutralization are highlighted in green. (0.30 MB TIF) [file ppat.1001028.s008.tif]

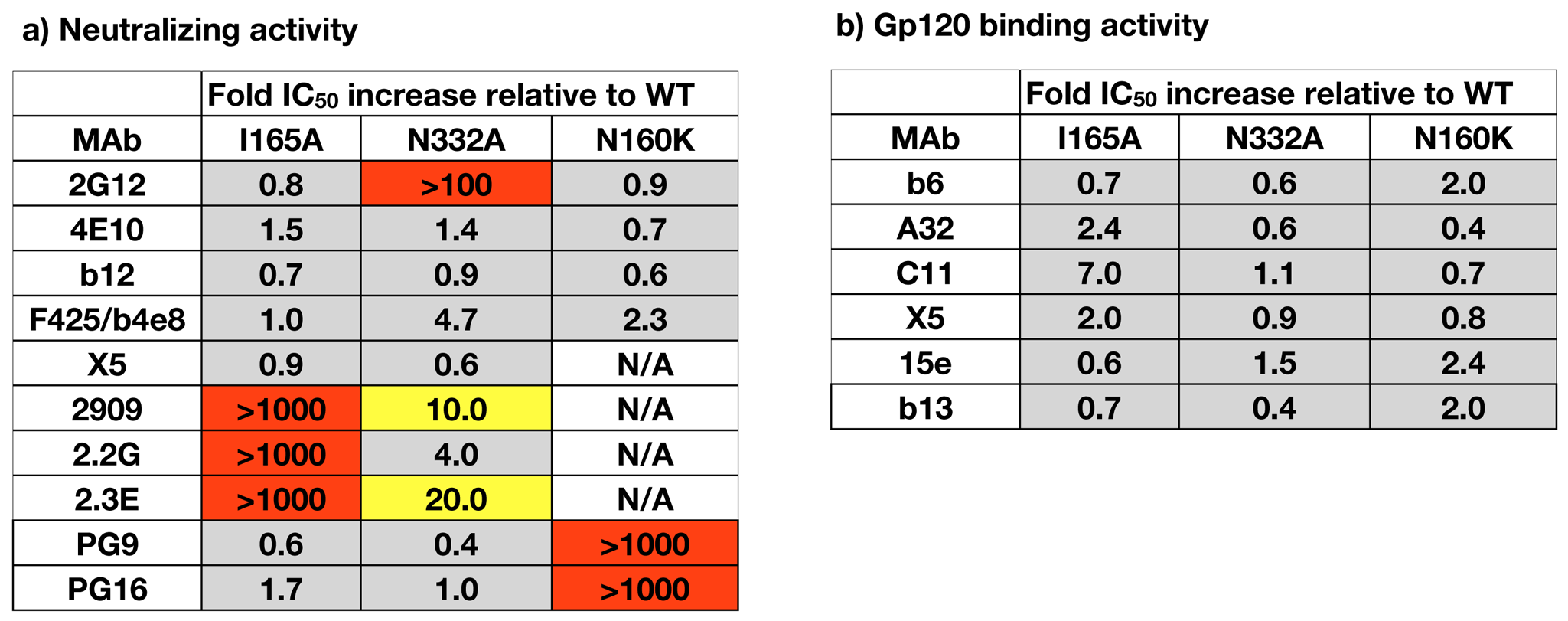

Supplement: Table S2 — Neutralizing and binding activity of MAbs to gp120 and pseudovirus variants relative to WT. aMAbs were tested for binding and neutralizing activity against gp120 and pseudovirus variants, respectively. A) All NAbs were tested against HIV-1 JR-CSF, except for 2909, 2.2G, and 2.3E, which were tested against HIV-1 SF162. B) ELISA binding assays were performed using gp120 captured from viral lysates, as described [50].c Binding and neutralizing activity is reported as fold increase in IC50 value relative to WT and was calculated using the equation (IC50 variant/IC50 WT). Boxes are color coded as follows: gray, 0–10 fold IC50 increase; yellow, 10–100 fold IC50 increase; red, >100 fold IC50 increase. (0.31 MB TIF) [file ppat.1001028.s009.tif]

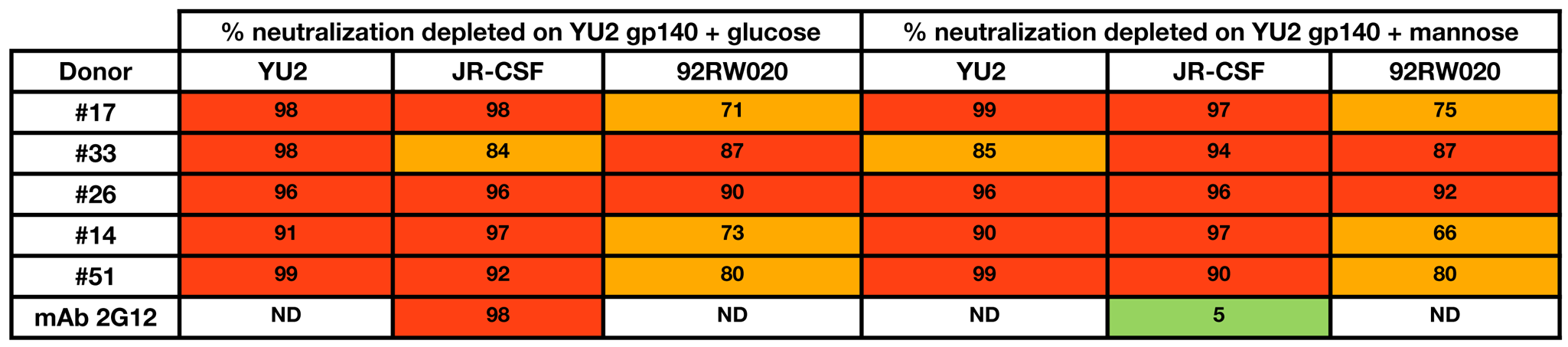

Supplement: Table S3 — Ability of mannose to inhibit gp140 depletion of serum neutralizing activity. Sera were tested for neutralizing activity after adsorption with gp140-coupled beads in the presence of 1M mannose or 1M glucose (control). The glycan-specific bNAb 2G12 was included as a positive control. % increase in IC50 was calculated using the equation = (1−(IC50 blank beads/IC50 antigen-coated beads))*100. Boxes are color coded as follows: Green, 0–45%, yellow, 45–65%; orange, 65–85%; red, 85–100%. (0.18 MB TIF) [file ppat.1001028.s010.tif]

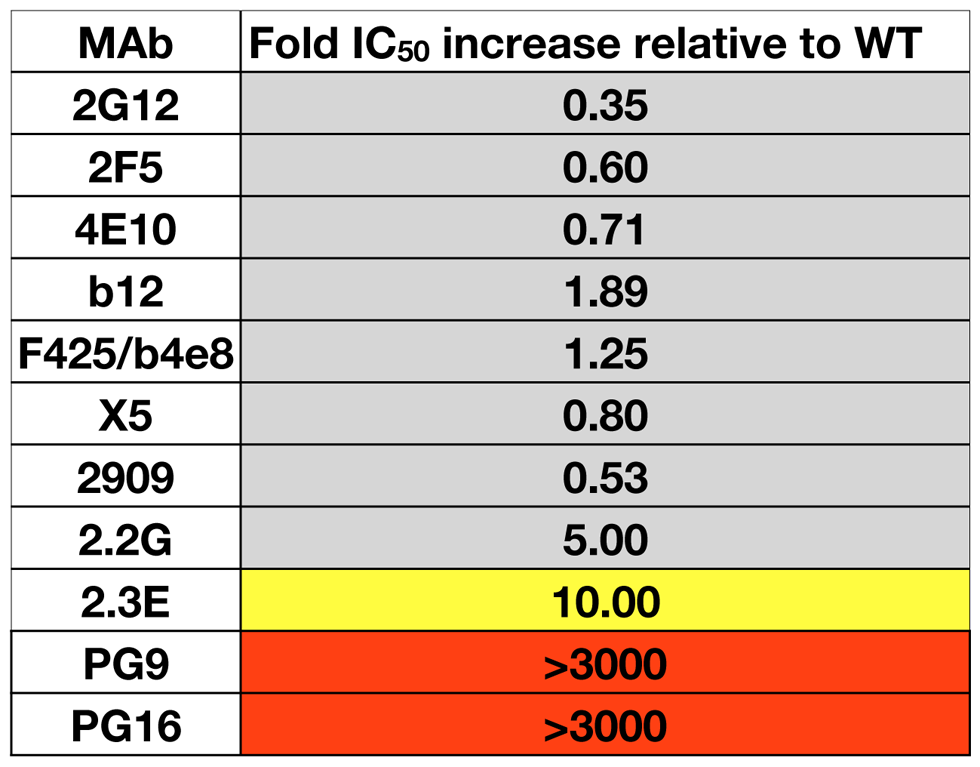

Supplement: Table S4 — Effect of kifunensine treatment on neutralization by NAbs. NAbs were tested for neutralizing activity against pseudoviruses treated with kifunensine. Neutralizing activity is reported as fold increase in IC50 value relative to WT and was calculated using the equation (IC50 variant/IC50 WT). All mAbs were tested against HIV-1 JR-CSF, except for 2909, 2.2G, and 2.3E, which were tested against HIV-1 SF162. Boxes are color coded as follows: gray, 0–10 fold IC50 increase; yellow, 10–100 fold IC50 increase; red, >100 fold IC50 increase. (0.14 MB TIF) [file ppat.1001028.s011.tif]
